# A facile approach to spiro[dihydrofuran-2,3'-oxindoles] via formal [4 + 1] annulation reaction of fused 1*H*-pyrrole-2,3-diones with diazooxindoles

**DOI:** 10.3762/bjoc.18.162

**Published:** 2022-11-10

**Authors:** Pavel A Topanov, Anna A Maslivets, Maksim V Dmitriev, Irina V Mashevskaya, Yurii V Shklyaev, Andrey N Maslivets

**Affiliations:** 1 Institute of Technical Chemistry, Ural Branch, Russian Academy of Sciences, Perm 614013, Russian Federation; 2 Department of Organic Chemistry, Faculty of Chemistry, Perm State University, Perm 614990, Russian Federation

**Keywords:** [4 + 1] annulation, catalyst-free, diazooxindole, 1*H*-pyrrole-2,3-diones, spirooxindole

## Abstract

There has been developed an easy synthetic approach to spiro[dihydrofuran-2,3'-oxindoles] via a highly diastereoselective formal [4 + 1] cycloaddition reaction of [*e*]-fused 1*H*-pyrrole-2,3-diones with diazooxindoles. The described novel heterocyclic systems are heteroanalogues of antimicrobial and antibiofilm fungal metabolites. The developed reaction represents the first example of involving 1*H*-pyrrole-2,3-diones fused at the [*e*]-side in a [4 + 1] annulation reaction.

## Introduction

**Figure 1:**
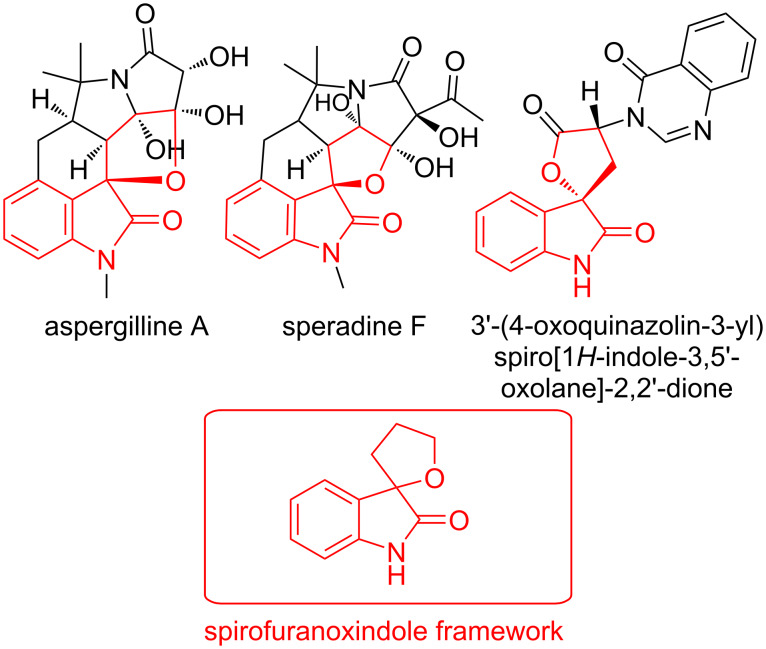
Selected examples of biologically active natural products bearing a spirofuranoxindole moiety.

Compounds with a spirooxindole scaffold have attracted the attention of researchers, which is demonstrated by the publication of several reviews of both the biological activities of compounds with the spirooxindole moiety [1–5], and methods for constructing spirooxindole systems by employing different approaches [6–12]. Cyclopiazonic acid derivatives such as aspergillins A–E [13] (Figure 1) and speradines C and F [14–15] are secondary metabolites of fungi, and include a furan fragment spiro-fused with 2-oxindole. Cyclopiamides I and J [16] were also isolated from the fungus *Penicillium commune* and contain a furan fragment spiro-annulated by 2-oxindole. These compounds exhibit anticancer [13] and antimicrobial [17] activities.

**Scheme 1:**
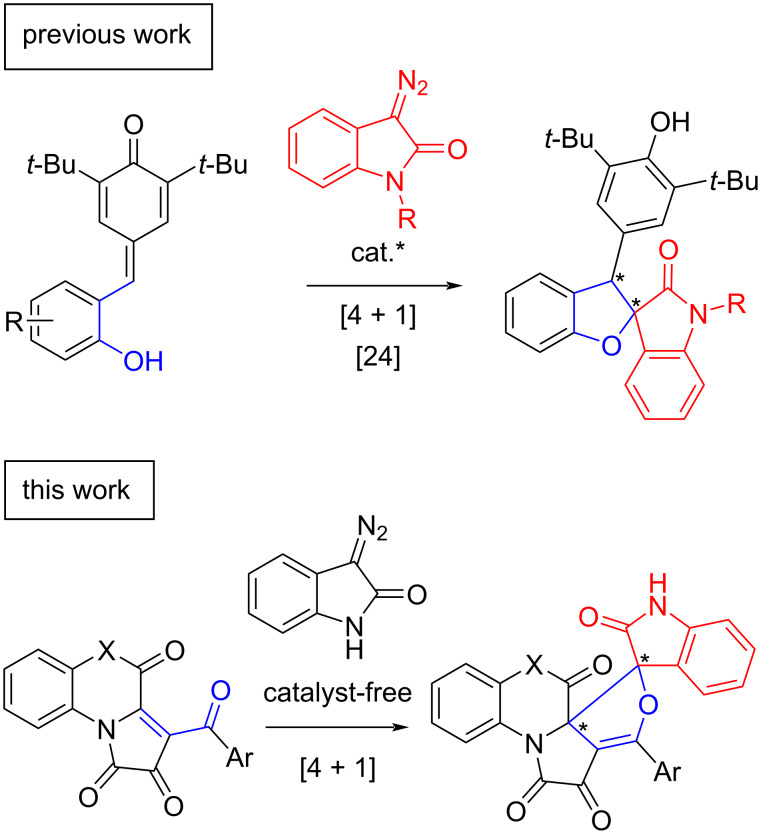
Synthesis of spiro[dihydrofuran-2,3'-oxindoles] from enones and diazooxindoles.

One of the expeditious methods for obtaining dihydrofurans is the cycloaddition reaction of diazo compounds to molecules containing an enone fragment. Cycloaddition reactions involving diazo and enone moieties are usually carried out using transition-metal catalysis [18–20], with catalyst-free reactions being carried out only with the participation of reactive unsubstituted diazomethane [21–23]. With diazooxindoles used as the diazo component, it is possible to obtain the desired spirofuranoxindoles. To date, only one method is known for obtaining spirofuranoxindoles from diazooxindole and an enone, where *p*-quinone methide acts as the enone, but the reaction requires the use of a catalyst [24] (Scheme 1).

Thus, in the present work, we report a simple, catalyst-free diastereoselective method for the synthesis of dihydrofurans spiro-annulated with an oxindole moiety for the first time. The essence of the method is the use of [*e*]-fused 1*H*-pyrrole-2,3-diones (FPDs) as the enone component in a formal [4 + 1] cycloaddition (Scheme 1).

## Results and Discussion

**Scheme 2:**
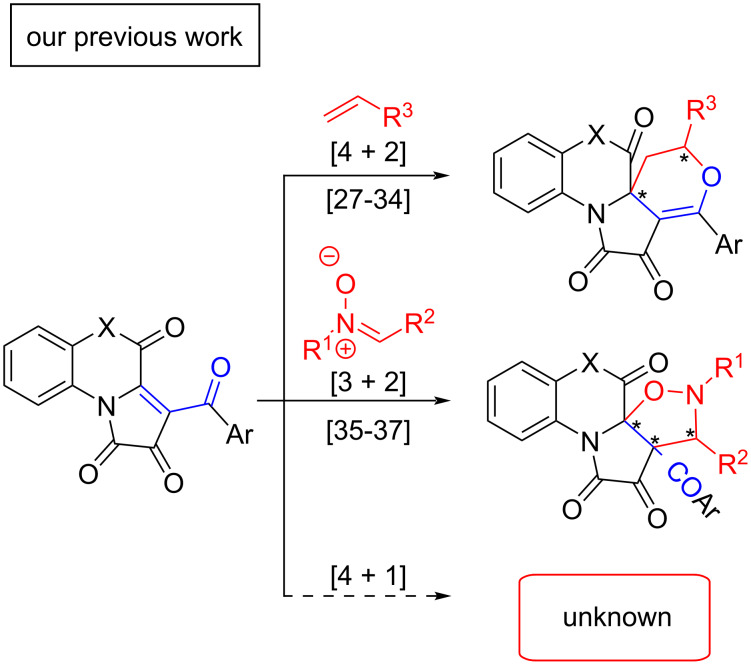
Cycloaddition reactions of [*e*]-fused 1*H*-pyrrole-2,3-diones.

FPDs are highly reactive compounds [25–26] containing an highly electrophilic enone fragment which facilitates the course of cycloaddition and nucleophilic addition reactions. In recent years, some types of cycloaddition reactions were investigated for FPDs: the [4 + 2] cycloaddition with alkenes resulting in pyran-annulated products [27–34] and the [3 + 2] cycloaddition with nitrones resulting in isoxazole-annulated products [35–37] (Scheme 2). However, formal [4 + 1] cycloaddition reactions for FPDs remain to be unknown.

**Scheme 3:**
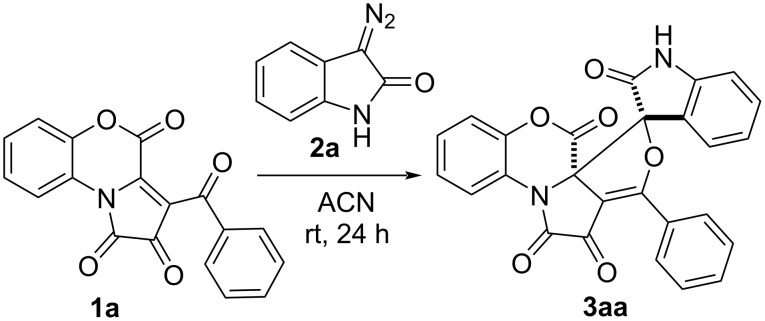
The model reaction of FPD **1a** and diazooxindole **2a**.

To evaluate the possibility of synthesizing the target spirooxindole compounds, we initially investigated a reaction of benzoxazine-containing FPD **1a** with diazooxindole **2a** in anhydrous acetonitrile at room temperature (Scheme 3). The reaction came to an end in 24 hours, with the color of the solution being turned from purple to red. The starting FPD **1a** is bright violet; thus, the disappearance of the violet color was used as an indicator of the reaction’s completion. Product **3aa** was isolated as yellow crystals in 73% yield and characterized by NMR, IR, and mass spectra, and single crystal X-ray analysis (CCDC 2201614). As evinced by the NMR data, only one diastereomer of product **3aa** was obtained. Contrary to the isoxazole-annulated products of a [3 + 2] cycloaddition of nitrones to FPDs [35], product **3aa** appears to be stable on storage in solution, which was confirmed by the fact that the NMR data remained unvaried after keeping the product in solution for one day.

Next, the conditions (Table 1) of the model reaction of FPD **1a** and diazooxindole **2a** were optimized.

**Table 1:** Reaction of FPD **1a** and diazooxindole **2a** in different solvents.^a^

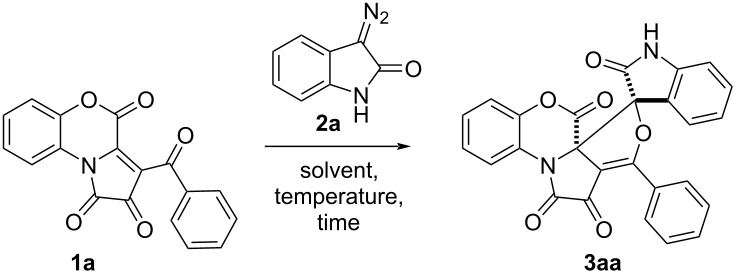			

Entry	Solvent	Temperature, °C	Yield^b^, %

1	toluene	25	3
2	chloroform	25	48
3	ethyl acetate	25	29
4	1,4-dioxane	25	9
5	DMSO	25	32
6	acetonitrile	25	82
7	acetonitrile	25	73^c^
8	acetonitrile	83	50^c^

^a^A suspension of FPD **1a** (100 µmol, 32.0 mg) and diazooxindole **2a** (100 µmol, 16.0 mg) in the corresponding solvent (1 mL) was stirred in an oven-dried closed microreaction vial for 24 hours; ^b^UPLC yield (the chromatograms were recorded immediately after sample preparation); ^c^isolated yield.

The best yield of product **3aа** (Table 1, entries 6 and 7) was obtained by the reaction performed in acetonitrile at room temperature, therefore, these conditions were taken as a standard for further reactions.

Next, the reagent scope of the reaction was explored by involving diazooxindoles **2a–d** into the reaction with FPD **1a** (Table 2).

**Table 2:** Reaction of FPD **1a** and diazooxindoles **2a–d**.^a^

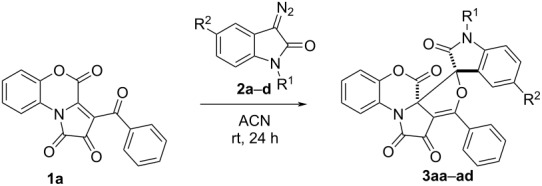							

Entry	Diazooxindole	Product	R^1^	R^2^	Time, h^b^	Yield^c^, %	dr^d^

1	**2a**	**3aa**	H	H	24	73	>99:1
2	**2b**	**3ab**	H	Br	168^e^	–^e^	–
3	**2b**	**3ab**	H	Br	6^f^	60	>99:1
4	**2c**	**3ac**	H	OMe	24	61	>99:1
5	**2d**	**3ad**	Bn	H	24	58	50:1

^a^A suspension of FPD **1a** (500 µmol, 160.0 mg) and diazooxindole **2a**–**d** (500 µmol) in acetonitrile (3 mL) was stirred in an oven-dried closed microreaction vial for the given time; ^b^reaction time was monitored by the disappearance of the dark violet color of FPD **1a**; ^c^isolated yield; ^d^ratio was determined by ^1^H NMR in isolated product; ^e^no disappearance of dark violet color of FPD **1a**, the reaction was monitored by UPLC-MS (the reaction not completed within a week); ^f^the reaction was carried out in refluxing solvent.

Compared to substrate **2a**, the presence of substituents in diazo compounds **2b–d** led to decreased reaction yields (Table 2, entries 2–5). The reaction with diazooxindole **2b** having an electron-withdrawing group (-Br) in the C(5) position (Table 2, entries 2 and 3) required additional heating to obtain the product **3ab**. On the other hand, the reaction of diazooxindoles **2c** and **2d** (Table 2, entries 4 and 5) bearing an EDG (electron-donating group) in positions N(1) (Bn-) or C(5) (MeO-) did not require heating and proceeded under conditions similar to the ones with unsubstituted diazooxindole **2a**.

Next, we investigated the substrate scope using different FPDs **2** (Table 3).

**Table 3:** Reaction of FPDs **1a**–**j** and diazooxindole **2a**.^a^

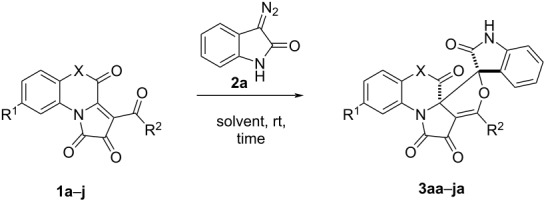							

Entry	Product	R^1^	R^2^	X	Time, h^b^	Yield^c^, %	dr^d^

1	**3aa**	H	Ph	O	24	73	>99:1
2	**3ba**	H	C_6_H_4_Cl-4	O	24	71	>99:1
3	**3ca**	H	C_6_H_4_Br-4	O	24	62	>99:1
4	**3da**	H	C_6_H_4_Me-4	O	24	73	>99:1
5	**3ea**	H	C_6_H_4_OMe-4	O	24	78	>99:1
6	**3fa**	H	C_6_H_4_NO_2_-4	O	24	48	>99:1
7	**3ga**	Cl	Ph	O	24	86	>99:1
8	**3ha**	H	C_6_H_4_Cl-4	NH	6^e^	70	50:1
9	**3ia**	H	Ph	N–Ph	6^e^	85	>99:1
10	**3ja**	H	C_6_H_4_Cl-4	N–Bn	6^e^	63	>99:1

^a^A suspension of FPD **1a–j** (500 µmol) and diazooxindole **2a** (500 µmol, 80.0 mg) in acetonitrile (3 mL) was stirred in an oven-dried closed microreaction vial; ^b^reaction time was monitored by the disappearance of the dark violet color of FPD **1**; ^c^isolated yield; ^d^ratio was determined by ^1^H NMR in isolated product; ^e^the reaction was carried out in refluxing solvent.

FPDs **1а**–**f** successfully reacted with diazooxindole **2а** under the previously developed conditions and gave good product yields (Table 3, entries 1–6). Neither the yield, nor the reaction rate were observed as being markedly affected by electron-donating or weak electron-withdrawing groups present in the aroyl substituent of FPDs **1**. However, the presence of a strong electron-withdrawing group (–NO_2_) in the aroyl fragment of the FPD **1f**, significantly decreased the yield of the target product **3fa** (Table 3, entry 6). The introduction of an electron-withdrawing group (–Cl) into the benzoxazine fragment of FPD **1g** increased the yield of the target reaction product, without affecting the reaction rate (Table 3, entry 7). Quinoxaline-annulated FPDs **1h**–**j** required heating, as these compounds reacted too slowly at room temperature (Table 3, entries 8–10). It should be noted that FPDs **1h**–**j** gave yields of the target products close to that of the products obtained from FPDs annulated with a benzoxazine fragment. The structures of products **3aa**, **3ab**, and **3ha** were approved by single crystal X-ray analysis (CCDC 2201614, CCDC 2201616, CCDC 2201615).

**Scheme 4:**
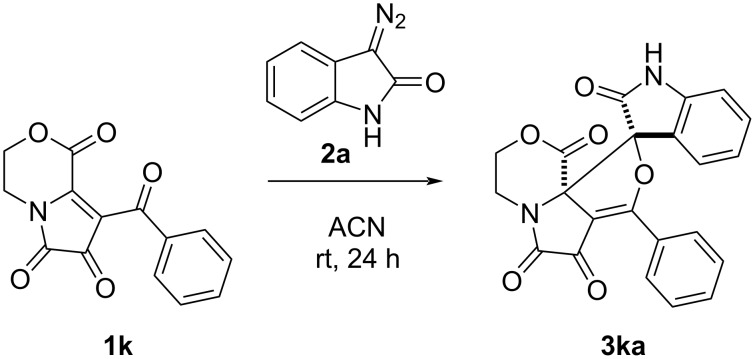
The reaction of FPD **1k** with diazooxindole **2a**.

We also decided to study the effect the benzo-annulated moiety in FPDs has on inducing the reaction. Under the same conditions, FPD **1k** containing a morpholine fragment (Scheme 4) was involved in the reaction with **2a** which gave the expected product **3ka** in a fairly good yield of 56% and dr 99:1. The characteristic signals in the NMR spectra of the products **3aa** and **3ka** appeared to be the same; thus, the structure of product **3ka** was ascertained to be similar to that of product **3aa**.

**Scheme 5:**
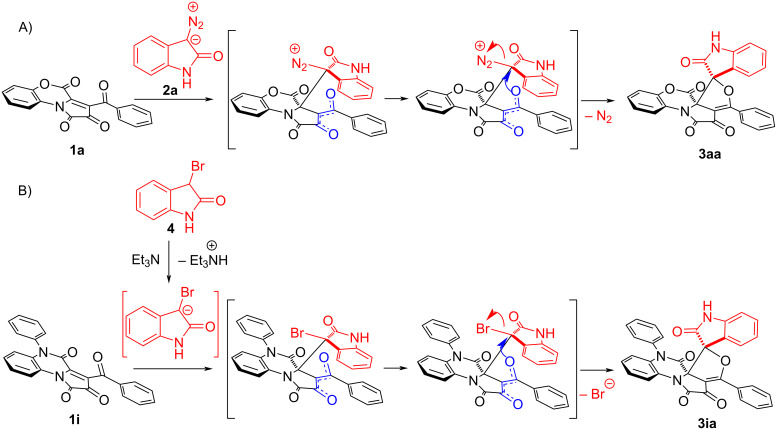
A) Plausible mechanism of formal [4 + 1] cycloaddition of FPDs **1** with diazooxindoles **2** (negative charge delocalization is colored in blue); B) plausible base-promoted reaction mechanism of FPD **1i** and 3-bromooxindole (**4**, negative charge delocalization is colored blue).

With the above observations and the reported literature [24] as a basis, the formation of spirofuranoxindoles **3aa–ka** was assumed as proceeding via two stages: (a) the nucleophilic Michael attack of the negatively charged [38] C(3) atom of diazooxindoles **2** at the C(3a) atom of FPDs **1** (Scheme 5), and (b) further intramolecular S_N_2 attack by the oxygen of the aroyl group with ensuing elimination of a nitrogen molecule. To verify our assumption, 3-bromooxindole (**4**) was involved in the reaction with FPD **1i** in the presence of 1.1 equiv of TEA. In this case, the base-promoted deprotonation of 3-bromooxindole (**4**) affords a highly nucleophilic intermediate, which undergoes Michael addition to FPD **1i**, followed by intramolecular S_N_2 attack [39–43] by the oxygen of the aroyl group (Scheme 5) to give the same diastereomer **3ia** with a good 54% yield.

## Conclusion

To conclude, we have developed a facile synthetic approach to spirofuranoxindoles **3** via the highly diastereoselective formal [4 + 1] cycloaddition reaction of FPDs **1** with diazooxindoles **2**. The obtained compounds **3** were found to be stable. Benzoxazine, quinoxaline, and morpholine FPDs were successfully involved into the reaction, with the modification of diazo compounds decreasing the reaction yield. The reaction time of the cycloaddition was found to be independent on substituents in the aroyl moiety of FPDs **1**. The described reaction is the first example of a catalyst-free formal [4 + 1] cycloaddition reaction of enones and complex diazo compounds. The synthesized compounds **3** have a pharmaceutically interesting fungal metabolites-like structure with a spiro[dihydrofuran-2,3'-oxindole] moiety.

## Supporting Information

File 1 Experimental part, compound characterization, and copies of NMR spectra.
