# Supplementary material for: A facile approach to spiro[dihydrofuran-2,3'-oxindoles] via formal [4 + 1] annulation reaction of fused 1H-pyrrole-2,3-diones with diazooxindoles
Source: Beilstein J Org Chem. 2022 Nov 10;18:1532–8. doi: 10.3762/bjoc.18.162 (PMC9663967; doi:10.3762/bjoc.18.162)
Supplement: File 1 — Experimental part, compound characterization, and copies of NMR spectra. [file Beilstein_J_Org_Chem-18-1532-s001.pdf]

BEILSTEIN JOURNAL OF ORGANIC CHEMISTRY

Supporting Information

for

A facile approach to spiro[dihydrofuran-2,3'-oxindoles] via formal [4 + 1] annulation reaction of fused 1*H*-pyrrole-2,3-diones with diazooxindoles

Pavel A. Topanov, Anna A. Maslivets, Maksim V. Dmitriev, Irina V. Mashevskaya, Yurii V. Shklyaev and Andrey N. Maslivets

Beilstein J. Org. Chem. **2022,** *18,* 1532–1538. doi:10.3762/bjoc.18.162

Experimental part, compound characterization, and copies of NMR spectra

License and Terms: This is a supporting information file under the terms of the Creative Commons Attribution License (https://creativecommons.org/licenses/by/4.0). Please note that the reuse, redistribution and reproduction in particular requires that the author(s) and source are credited and that individual graphics may be subject to special legal provisions.
The license is subject to the *Beilstein Journal of Organic Chemistry* terms and conditions: (https://www.beilstein-journals.org/bjoc/terms)

Table of contents

General information....S2

General procedure to compounds **3aa**–**ka**....S3

NMR charts of compounds **3aa**–**ka**....S9

NMR charts of compound **3aa** after dissolution and 24 h later....S39

X-Ray crystal data of CCDC 2201614 (**3aa**)....S41

X-Ray crystal data of CCDC 2201616 (**3ab**)....S43

X-Ray crystal data of CCDC 2201615 (**3ha**)....S45

References....S47

General information

^{1}H and ^{13}C NMR spectra were acquired on a Bruker Avance-III spectrometer (400 and 100 MHz, respectively) in DMSO-d_6 using the residual solvent signals (in ^{1}H NMR 2.50 ppm for DMSO-d_6; in ^{13}C NMR 39.51 ppm for DMSO-d_6) as internal standards. Melting points were measured on a Khimlabpribor PTP apparatus or a Mettler Toledo MP70 apparatus. The reaction conditions were optimized using UPLC–UV–MS [Waters ACQUITY UPLC I-Class system; Acquity UPLC BEH C18 column, grain size of 1.7 μm; acetonitrile/water as eluents; flow rate of 0.6 mL/min; ACQUITY UPLC PDA eλ Detector (wavelength range of 230–780 nm); Xevo TQD mass detector; electrospray ionization; positive and negative ion detection; ion source temperature of 150 °C; capillary voltage of 3500–4000 V; cone voltage of 20–70 V; vaporizer temperature of 200 °C]. Thin-layer chromatography (TLC) was performed on Merck silica gel 60 F_{254} plates using EtOAc/toluene 1:3 (v/v), acetic acid/EtOAc/toluene 1:8:24 (v/v) acetic acid, toluene, EtOAc as eluents, and the spots were visualized with UV light (254 and 365 nm) or by treating the plate with iodine vapor. HRMS were recorded on Bruker MicroTOF (ESI+), acetonitrile and methanol were used as solvents. The single crystal X-ray analyses of compounds **3aa**, **3ab**, and **3ha** were performed on an Xcalibur Ruby diffractometer (Agilent Technologies). The empirical absorption correction was introduced by multi-scan method using SCALE3 ABSPACK algorithm.[1] Using OLEX2[2], the structures were solved with the SUPERFLIP[3] program or with the olex2.solve[4] program and refined by the full-matrix least-squares minimization in the anisotropic approximation for all non-hydrogen atoms with the SHELXL[5] program. Hydrogen atoms bound to carbon were positioned geometrically and refined using a riding model. The hydrogen atoms of NH groups were refined freely with isotropic displacement parameters.

Starting FPDs **1a–k** were obtained according to reported procedures[6] from oxalyl chloride (purchased from commercial vendors) and heterocyclic enamines (obtained according to reported procedures[6] from commercially available reagents).

Diazooxindoles **2a–d** and 3-bromooxindole (**4**) were obtained according to reported procedures[7] from commercially available reagents.

Toluene, and 1,4-dioxane were distilled over Na before use. Ethyl acetate, chloroform, and acetone were distilled over P_2O_5 before the use. Acetonitrile, DMSO, DMF were dried over molecular sieves 4 Å before use. All other solvents and reagents were purchased from commercial vendors and were used as received.

Procedures involving FPDs **1a–k** were carried out in oven-dried glassware.

General procedure to compounds 3aa–ka

A suspension of the corresponding FPD **1** (500 µmol) and diazooxindole **2** (500 µmol) in 3 mL of acetonitrile was vigorously stirred at room temperature for 24 hours or at 83 °C for 3–5 hours (for FPDs **1h**–**j**) (until the disappearance of the dark violet color of FPD **1**). Then, the resulting yellow or pale-yellow precipitate was filtered off and washed with acetonitrile (2 × 1 mL) to afford the desired compounds **3**. Product **3ja** was additionally recrystallized from 1,4-dioxane.

Control experiment:

A suspension of the FPD **1i** (500 µmol) and 3-bromoxindole (**4**, 500 µmol) in 3 mL of acetonitrile was stirred at room temperature for 2 minutes and then 1.1 equiv of TEA was added in one portion. The mixture was stirred for 5 minutes (until the disappearance of the dark violet color of FPD **1i**). Then, the resulting yellow precipitate was filtered off and washed with acetonitrile (2 × 1 mL) to afford the desired compound **3ia**.

((5*R,5a*R**)-3-Phenyl-6*H*-spiro[benzo[*b*]furo[3',4':2,3]pyrrolo[1,2-*d*][1,4]oxazine-5,3'-indoline]-1,2,2',6-tetraone (3aa)**

Yield: 165 mg (73%); yellow solid; mp 246–247°C (decomp.).

^{1}H NMR (400 MHz, DMSO-d_6) δ 10.83 (s, 1H), 8.24 (d, *J* = 7.3 Hz, 2H), 7.86 (t, *J* = 7.5 Hz, 1H), 7.72 (t, *J* = 7.7 Hz, 2H), 7.30 (qd, *J* = 5.3, 4.8, 2.2 Hz, 3H), 7.06 (ddd, *J* = 8.6, 6.1, 2.7 Hz, 1H), 7.00 – 6.90 (m, 3H), 6.65 (d, *J* = 7.8 Hz, 1H).

^{13}C NMR (101 MHz, DMSO-d_6) δ 173.28, 169.07, 167.07, 160.47, 158.64, 143.17, 141.48, 135.55, 132.61, 129.99 (2C), 129.34 (2C), 127.89, 125.69, 124.60, 124.11, 122.77, 121.81, 121.44, 121.28, 116.67, 110.77, 105.02, 91.14, 70.63.

MS (ESI+): m/z calcd for $C_{26}H_{14}N_2O_6$+MeOH+Na^+: 505.1006 [M+ MeOH+Na^+]; found: 505.1011.

(5*R,5a*R**)-5'-Bromo-3-phenyl-6*H*-spiro[benzo[*b*]furo[3',4':2,3]pyrrolo[1,2-*d*][1,4]oxazine-5,3'-indoline]-1,2,2',6-tetraone (3ab)**

Yield: 159 mg (60%); yellow solid; mp 225–226 °C (decomp.).

^{1}H NMR (400 MHz, DMSO-d_6) δ 11.00 (s, 1H), 8.23 (d, *J* = 7.5 Hz, 2H), 7.85 (t, *J* = 7.4 Hz, 1H), 7.72 (t, *J* = 7.7 Hz, 2H), 7.50 – 7.44 (m, 1H), 7.36 (d, *J* = 1.5 Hz, 1H), 7.29 (d, *J* = 5.4 Hz, 2H), 7.12 – 7.06 (m, 1H), 6.98 (d, *J* = 7.8 Hz, 1H), 6.59 (d, *J* = 8.4 Hz, 1H).

^{13}C NMR (101 MHz, DMSO-d_6) δ 173.14, 169.01, 166.82, 160.44, 158.35, 143.16, 140.82, 135.44, 135.27, 130.09 (2C), 129.25 (2C), 127.92, 127.27, 125.73, 124.75, 123.40, 121.88, 121.44, 116.72, 114.04, 112.63, 104.46, 90.51, 71.18.

MS (ESI+): m/z calcd for $C_{26}H_{13}BrN_2O_6$+MeOH+Na^+: 583.0111 [M+ MeOH+Na^+]; found: 583.0117.

(5*R,5a*R**)-5'-Methoxy-3-phenyl-6*H*-spiro[benzo[*b*]furo[3',4':2,3]pyrrolo[1,2-*d*][1,4]oxazine-5,3'-indoline]-1,2,2',6-tetraone (3ac)**

Yield: 146 mg (61%); yellow solid; mp 255–256°C (decomp.).

^{1}H NMR (400 MHz, DMSO-d_6) δ 10.67 (s, 1H), 8.23 (d, *J* = 7.5 Hz, 2H), 7.86 (t, *J* = 7.4 Hz, 1H), 7.73 (t, *J* = 7.7 Hz, 2H), 7.33 – 7.23 (m, 2H), 7.10 – 7.05 (m, 1H), 7.00 (d, *J* = 7.6 Hz, 1H), 6.90 (dd, *J* = 8.6, 2.4 Hz, 1H), 6.58 (d, *J* = 8.6 Hz, 1H), 6.51 (d, *J* = 2.3 Hz, 1H), 3.60 (s, 3H).

^{13}C NMR (101 MHz, DMSO-d_6) δ 173.28, 168.96, 167.20, 160.49, 158.59, 155.09, 143.15, 135.54, 134.73, 130.06 (2C), 129.32 (2C), 127.85, 125.71, 124.64, 122.26, 121.77, 121.45, 117.35, 116.66, 111.50, 110.93, 104.85, 91.27, 70.68, 55.79.

MS (ESI+): m/z calcd for $C_{27}H_{16}N_2O_7$+Na^+: 503.0850 [M+Na^+]; found: 503.0860.

(5*R,5a*R**)-1'-Benzyl-3-phenyl-6*H*-spiro[benzo[*b*]furo[3',4':2,3]pyrrolo[1,2-*d*][1,4]oxazine-5,3'-indoline]-1,2,2',6-tetraone (3ad)**

Yield: 157 mg (58%); yellow solid; mp 240–241°C (decomp.).

^{1}H NMR (400 MHz, DMSO-d_6) δ 8.28 – 8.22 (m, 2H), 7.89 – 7.82 (m, 1H), 7.72 (t, *J* = 7.8 Hz, 2H), 7.36 – 7.23 (m, 5H), 7.18 (dd, *J* = 8.3, 1.3 Hz, 1H), 7.05 (dtd, *J* = 21.0, 7.6, 1.1 Hz, 3H), 6.97 (dd, *J* = 8.0, 1.6 Hz, 1H), 6.92 – 6.86 (m, 2H), 6.75 (d, *J* = 7.9 Hz, 1H), 4.80 (d, *J* = 15.9 Hz, 1H), 4.64 (d, *J* = 15.9 Hz, 1H), minor product: 5.00 (d, *J* = 15.8 Hz, 1H).

^{13}C NMR (101 MHz, DMSO-d_6) δ 173.13, 167.70, 166.87, 160.77, 158.40, 142.95, 142.02, 135.62, 134.60, 132.70, 130.01 (2C), 129.36 (2C), 128.67 (2C), 127.93, 127.41, 126.75 (2C), 125.64, 124.69, 124.06, 123.70, 121.98, 121.23, 120.79, 116.81, 110.52, 105.33, 90.51, 70.59, 43.39.

MS (ESI+): m/z calcd for $C_{33}H_{20}N_2O_6$+Na^+: 563.1213 [M+Na^+]; found: 563.1215.

(5*R,5a*R**)-3-(4-Chlorophenyl)-6*H*-spiro[benzo[*b*]furo[3',4':2,3]pyrrolo[1,2-*d*][1,4]oxazine-5,3'-indoline]-1,2,2',6-tetraone (3ba)**

Yield: 172 mg (71%); yellow solid; mp 225–226 °C (decomp.).

^{1}H NMR (400 MHz, DMSO-d_6) δ 10.83 (s, 1H), 8.23 (d, *J* = 8.6 Hz, 2H), 7.81 (d, *J* = 8.6 Hz, 2H), 7.28 (q, *J* = 6.0, 5.1 Hz, 3H), 7.04 (ddd, *J* = 18.3, 10.1, 4.8 Hz, 2H), 6.97 – 6.89 (m, 2H), 6.64 (d, *J* = 7.8 Hz, 1H).

^{13}C NMR (101 MHz, DMSO-d_6) δ 173.38, 168.98, 165.68, 160.31, 158.53, 143.17, 141.46, 140.43, 132.66, 131.49 (2C), 129.65 (2C), 127.95, 124.64, 124.50, 124.24, 122.78, 121.84, 121.40, 121.21, 116.69, 110.75, 105.40, 91.34, 70.65.

MS (ESI+): m/z calcd for $C_{26}H_{13}ClN_2O_6$+MeOH+Na^+: 539.0616 [M+ MeOH+Na^+]; found: 539.0638.

(5*R,5a*R**)-3-(4-Bromophenyl)-6*H*-spiro[benzo[*b*]furo[3',4':2,3]pyrrolo[1,2-*d*][1,4]oxazine-5,3'-indoline]-1,2,2',6-tetraone (3ca)**

Yield: 159 mg (62%); yellow solid; mp 230–231 °C (decomp.).

^{1}H NMR (400 MHz, DMSO-d_6) δ 10.83 (s, 1H), 8.14 (d, *J* = 8.5 Hz, 2H), 7.95 (d, *J* = 8.5 Hz, 2H), 7.29 (q, *J* = 7.3, 5.2 Hz, 3H), 7.04 (ddd, *J* = 18.9, 10.4, 4.8 Hz, 2H), 6.98 – 6.88 (m, 2H), 6.64 (d, *J* = 7.8 Hz, 1H).

^{13}C NMR (101 MHz, DMSO-d_6) δ 173.38, 168.97, 165.81, 160.29, 158.49, 143.16, 141.45, 132.65, 132.60 (2C), 131.44 (2C), 129.77, 127.94, 124.80, 124.63, 124.24, 122.77, 121.83, 121.39, 121.20, 116.69, 110.74, 105.46, 91.33, 70.65.

MS (ESI+): m/z calcd for $C_{26}H_{13}BrN_2O_6$+MeOH+Na$^+$: 583,0111 [M+Na$^+$]; found: 583.0126.

(5*R,5a*R**)-3-(4-Methylphenyl)-6*H*-spiro[benzo[*b*]furo[3',4':2,3]pyrrolo[1,2-*d*][1,4]oxazine-5,3'-indoline]-1,2,2',6-tetraone (3da)**

Yield: 170 mg (73%); yellow solid; mp 247–248°C (decomp.).

^{1}H NMR (400 MHz, DMSO-d_6) δ 10.82 (s, 1H), 8.14 (d, *J* = 8.1 Hz, 2H), 7.54 (d, *J* = 8.1 Hz, 2H), 7.31 – 7.27 (m, 3H), 7.05 (ddd, *J* = 8.6, 6.1, 2.6 Hz, 1H), 7.00 – 6.88 (m, 3H), 6.65 (d, *J* = 7.8 Hz, 1H), 2.48 (s, 3H).

^{13}C NMR (101 MHz, DMSO-d_6) δ 173.15, 169.11, 167.41, 160.65, 158.76, 147.02, 143.17, 141.50, 132.59, 130.10 (2C), 129.98 (2C), 127.86, 124.59, 123.95, 123.08, 122.76, 121.77, 121.48, 121.31, 116.67, 110.80, 104.33, 91.02, 70.58, 21.56.

MS (ESI+): m/z calcd for $C_{27}H_{16}N_2O_6$+MeOH+Na$^+$: 519.1162 [M+Na$^+$]; found: 519.1173.

(5*R,5a*R**)-3-(4-Methoxyphenyl)-6*H*-spiro[benzo[*b*]furo[3',4':2,3]pyrrolo[1,2-*d*][1,4]oxazine-5,3'-indoline]-1,2,2',6-tetraone (3ea)**

Yield: 187 mg (78%); yellow solid; mp 247–248 °C (decomp.).

^{1}H NMR (400 MHz, DMSO-d_6) δ 10.81 (s, 1H), 8.23 (d, *J* = 8.8 Hz, 2H), 7.34 – 7.22 (m, 5H), 7.05 (td, *J* = 7.0, 6.1, 2.6 Hz, 1H), 6.98 – 6.92 (m, 2H), 6.88 (d, *J* = 7.4 Hz, 1H), 6.65 (d, *J* = 7.8 Hz, 1H), 3.94 (s, 3H).

^{13}C NMR (101 MHz, DMSO-d_6) δ 172.91, 169.17, 167.50, 165.47, 160.97, 158.95, 143.15, 141.51, 132.76 (2C), 132.53, 127.76, 124.56, 123.84, 122.73, 121.71, 121.55, 121.34, 118.13, 116.64, 115.10 (2C), 110.79, 103.00, 90.79, 70.56, 55.97.

MS (ESI+): m/z calcd for $C_{27}H_{16}N_2O_7$+Na$^+$: 503.0850 [M+Na$^+$]; found: 503.0850.

(5*R,5a*R**)-3-(4-Nitrophenyl)-6*H*-spiro[benzo[*b*]furo[3',4':2,3]pyrrolo[1,2-*d*][1,4]oxazine-5,3'-indoline]-1,2,2',6-tetraone (3fa)**

Yield: 119 mg (48%); yellow solid; mp 234–236°C (decomp.).

^{1}H NMR (400 MHz, DMSO-d_6) δ 10.87 (s, 1H), 8.56 – 8.38 (m, 4H), 7.31 (q, *J* = 7.8 Hz, 3H), 7.12 (d, *J* = 7.5 Hz, 1H), 7.10 – 7.02 (m, 1H), 7.00 – 6.89 (m, 2H), 6.64 (d, *J* = 7.8 Hz, 1H).

^{13}C NMR (101 MHz, DMSO-d_6) δ 173.60, 168.90, 163.84, 159.91, 158.26, 150.75, 143.19, 141.45, 132.77, 130.97 (2C), 130.79, 128.09, 124.72, 124.56, 124.30 (2C), 122.80, 121.94, 121.30, 121.16, 116.75, 110.75, 107.52, 91.72, 70.77.

MS (ESI+): m/z calcd for $C_{26}H_{13}N_3O_8$+MeOH+Na$^+$: 550,0857 [M+ MeOH+Na$^+$]; found: 550.0875.

(5*R,5a*R**)-10-Chloro-3-phenyl-6*H*-spiro[benzo[*b*]furo[3',4':2,3]pyrrolo[1,2-*d*][1,4]oxazine-5,3'-indoline]-1,2,2',6-tetraone (3ga)**

Yield: 208 mg (86%); yellow solid; mp 259–260°C (decomp.).

^{1}H NMR (400 MHz, DMSO-d_6) δ 10.89 (s, 1H), 8.24 (d, *J* = 7.6 Hz, 2H), 7.86 (t, *J* = 7.4 Hz, 1H), 7.72 (t, *J* = 7.6 Hz, 2H), 7.35 (d, *J* = 12.6 Hz, 3H), 7.04 – 6.95 (m, 2H), 6.88 (s, 1H), 6.70 (d, *J* = 7.8 Hz, 1H).

^{13}C NMR (101 MHz, DMSO-d_6) δ 172.79, 169.12, 167.57, 160.35, 158.06, 142.10, 141.42, 135.69, 132.67, 130.10 (2C), 129.36 (2C), 128.13, 127.74, 125.59, 124.33, 122.92, 122.45, 121.20, 121.07, 118.51, 110.83, 104.46, 91.20, 70.54.

MS (ESI+): m/z calcd for $C_{26}H_{13}ClN_2O_6$+MeOH+Na$^+$: 539,0616 [M+ MeOH+Na$^+$]; found: 539.0623.

(5*R,5a*R**)-3-(4-Chlorophenyl)spiro[furo[3',4':2,3]pyrrolo[1,2-*a*]quinoxaline-5,3'-indoline]-1,2,2',6(7*H*)-tetraone ACN solvate 1:1 (3ha)**

Yield: 184 mg (70%); yellow solid; mp 252–253°C (decomp.).

^{1}H NMR (400 MHz, DMSO-d_6) δ 11.06 (s, 1H), 10.65 (s, 1H), 8.24 (d, *J* = 8.7 Hz, 2H), 7.80 (d, *J* = 8.7 Hz, 2H), 7.30 – 7.21 (m, 1H), 7.19 – 7.14 (m, 1H), 7.01 (d, *J* = 8.0 Hz, 1H), 6.98 – 6.90 (m, 2H), 6.86 – 6.81 (m, 1H), 6.77 (d, *J* = 6.8 Hz, 1H), 6.60 (d, *J* = 7.8 Hz, 1H), minor product: 10.98 (s, 1H), 10.76 (s, 1H), 7.92 – 7.86 (m, 2H).

^{13}C NMR (101 MHz, DMSO-d_6) δ 173.81, 169.21, 165.37, 160.83, 160.66, 141.43, 140.15, 132.22, 131.42 (2C), 130.40, 129.55 (2C), 127.41, 124.80, 123.79, 122.40, 122.20, 122.13, 121.62, 121.39, 115.66, 110.50, 106.10, 92.13, 71.19.

MS (ESI+): m/z calcd for $C_{26}H_{14}ClN_3O_5$+MeOH+Na$^+$: 538,0776 [M+MeOH+Na$^+$]; found: 538.0764.

(5*R,5a*R**)-3,7-Diphenyl-6*H*-spiro[benzo[*b*]furo[3',4':2,3]pyrrolo[1,2-*d*][1,4]oxazine-5,3'-indoline]-1,2,2',6-tetraone (3ia)**

Yield: 223 mg (85%); yellow solid; mp 254–255°C (decomp.).

^{1}H NMR (400 MHz, DMSO-d_6) δ 10.82 (s, 1H), 8.26 – 8.22 (m, 2H), 7.84 (tt, *J* = 7.0, 1.2 Hz, 1H), 7.72 (t, *J* = 7.7 Hz, 2H), 7.58 (t, *J* = 7.4 Hz, 2H), 7.52 – 7.47 (m, 1H), 7.39 (br s,

2H) 7.31 – 7.26 (m, 1H), 7.12 – 7.07 (m, 1H), 7.02 – 6.95 (m, 2H), 6.89 (td, *J* = 7.7, 1.2 Hz, 1H), 6.84 (dd, *J* = 7.9, 1.5 Hz, 1H), 6.62 (d, *J* = 7.8 Hz, 1H), 6.35 (dd, *J* = 8.3, 0.9 Hz, 1H).

^{13}C NMR (101 MHz, DMSO-d_6) δ 173.87, 169.50, 166.83, 160.51, 160.32, 141.45, 137.08, 135.35, 133.24, 132.31, 129.95 (2C), 129.56 (2C), 129.30 (2C), 128.74 (2C), 128.57, 127.17, 125.95, 123.77, 122.88, 122.63, 122.52, 122.17, 121.88, 116.72, 110.62, 105.87, 91.73, 71.06.

MS (ESI+): m/z calcd for $C_{32}H_{19}N_3O_5$+Na^+: 548.1217 [M+Na^+]; found: 548.1208.

Control experiment:

Yield: 142 mg (54%); yellow solid; mp 254–255°C (decomp.).

^{1}H NMR (400 MHz, DMSO-d_6) δ 10.81 (s, 1H), 8.27 – 8.22 (m, 2H), 7.84 (tt, *J* = 7.0, 1.2 Hz, 1H), 7.72 (t, *J* = 7.7 Hz, 2H), 7.58 (t, *J* = 7.6 Hz, 2H), 7.52 – 7.47 (m, 1H), 7.39 (br s, 2H), 7.31 – 7.26 (m, 1H), 7.12 – 7.07 (m, 1H), 7.02 – 6.95 (m, 2H), 6.89 (dd, *J* = 15.3, 1.2 Hz, 1H), 6.83 (dd, *J* = 7.9, 1.5 Hz, 1H), 6.62 (d, *J* = 7.8 Hz, 1H), 6.34 (dd, *J* = 8.3, 0.9 Hz, 1H).

^{13}C NMR (101 MHz, DMSO-d_6) δ 173.85, 169.47, 166.80, 160.48, 160.29, 141.43, 137.06, 135.32, 133.23, 132.29, 129.93 (2C), 129.54 (2C), 129.28 (2C), 128.73 (2C), 128.54, 127.14, 125.94, 123.76, 122.86, 122.60, 122.51, 122.16, 121.85, 116.69, 110.59, 105.85, 91.71, 71.04.

(5*S,5a*S**)-7-Benzyl-3-(4-chlorophenyl)spiro[furo[3',4':2,3]pyrrolo[1,2-*a*]quinoxaline-5,3'-indoline]-1,2,2',6(7*H*)-tetraone 1:1 dioxane solvate (3ja)**

Yield: 210 mg (63%); yellow solid; mp 258–260 °C (decomp.).

^{1}H NMR (400 MHz, DMSO-d_6) δ 10.68 (s, 1H), 8.26 (d, *J* = 8.6 Hz, 2H), 7.81 (d, *J* = 8.6 Hz, 2H), 7.38 (d, *J* = 7.4 Hz, 2H), 7.27 (td, *J* = 7.3, 3.1 Hz, 3H), 7.22 (d, *J* = 7.2 Hz, 1H), 7.14 (d, *J* = 7.0 Hz, 2H), 6.96 (dt, *J* = 14.8, 7.1 Hz, 2H), 6.88 – 6.83 (m, 1H), 6.80 (d, *J* = 7.4 Hz, 1H), 6.60 (d, *J* = 7.8 Hz, 1H), 5.40 (d, *J* = 16.5 Hz, 1H), 4.93 (d, *J* = 16.4 Hz, 1H).

^{13}C NMR (101 MHz, DMSO-d_6) δ 173.83, 169.20, 165.44, 161.39, 160.28, 141.49, 140.20, 136.04, 132.31, 131.45 (2C), 130.85, 129.59 (2C), 128.19 (2C), 127.33, 127.21 (2C), 126.97, 124.78, 123.76, 122.74, 122.70, 122.53, 122.03, 121.80, 116.15, 110.57, 106.58, 91.80, 71.10, 45.63.

MS (ESI+): m/z calcd for $C_{33}H_{20}ClN_3O_5$+Na^+: 596,0983 [M+Na^+]; found: 596.0986.

(10*R,10a*R**)-8-Phenyl-3,4-dihydro-1*H*-spiro[furo[3',4':2,3]pyrrolo[2,1-*c*][1,4]oxazine-10,3'-indoline]-1,2',6,7-tetraone (3ka)**

Yield: 113 mg (56%); yellow solid; mp 217–218°C (decomp.).

^{1}H NMR (400 MHz, DMSO-d_6) δ 11.35 (s, 1H), 8.19 (d, *J* = 8.4 Hz, 2H), 7.82 (t, *J* = 7.5 Hz, 1H), 7.68 (t, *J* = 7.8 Hz, 2H), 7.48 – 7.42 (m, 1H), 7.09 – 6.94 (m, 3H), 4.61 (ddd, *J* = 12.9, 5.8, 2.0 Hz, 1H), 3.99 (td, *J* = 12.7, 12.3, 4.3 Hz, 1H), 3.74 (ddd, *J* = 13.2, 11.7, 6.0 Hz, 1H), 2.94 (ddd, *J* = 13.4, 4.1, 2.0 Hz, 1H).

^{13}C NMR (101 MHz, DMSO-d_6) δ 173.55, 169.72, 165.82, 164.54, 164.05, 141.81, 135.45, 132.93, 129.86 (2C), 129.29 (2C), 125.64, 125.01, 123.39, 121.98, 111.66, 108.44, 90.39, 72.14, 63.54 (2C).

MS (ESI+): m/z calcd for $C_{22}H_{14}N_2O_6+Na^+$: 425,0744 [$M+Na^+$]; found: 425.0747.

NMR charts of compounds 3aa–ka

3aa ^{1}H (400 MHz, DMSO-d_6)

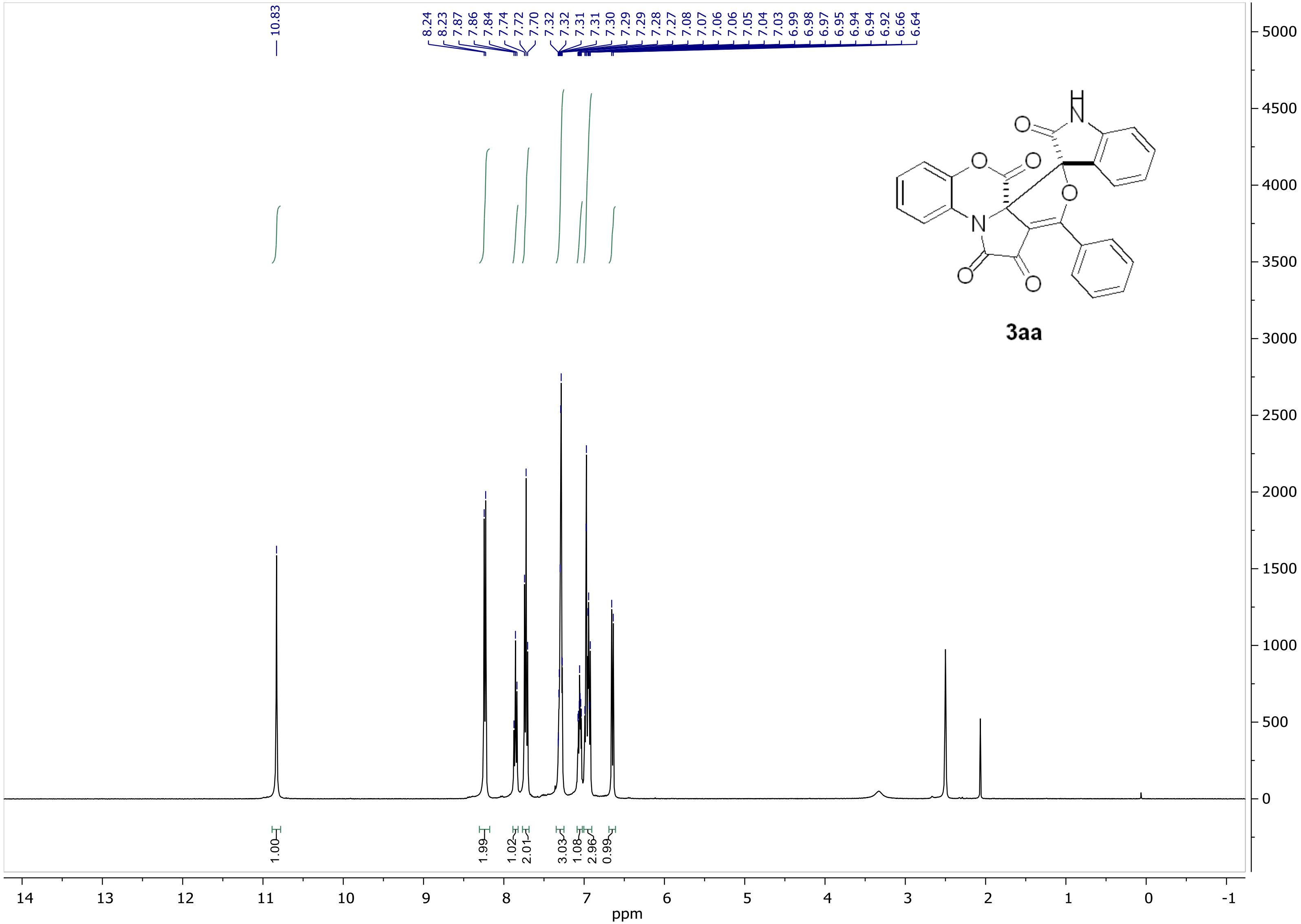

3aa ^{13}C (101 MHz, DMSO-d_6)

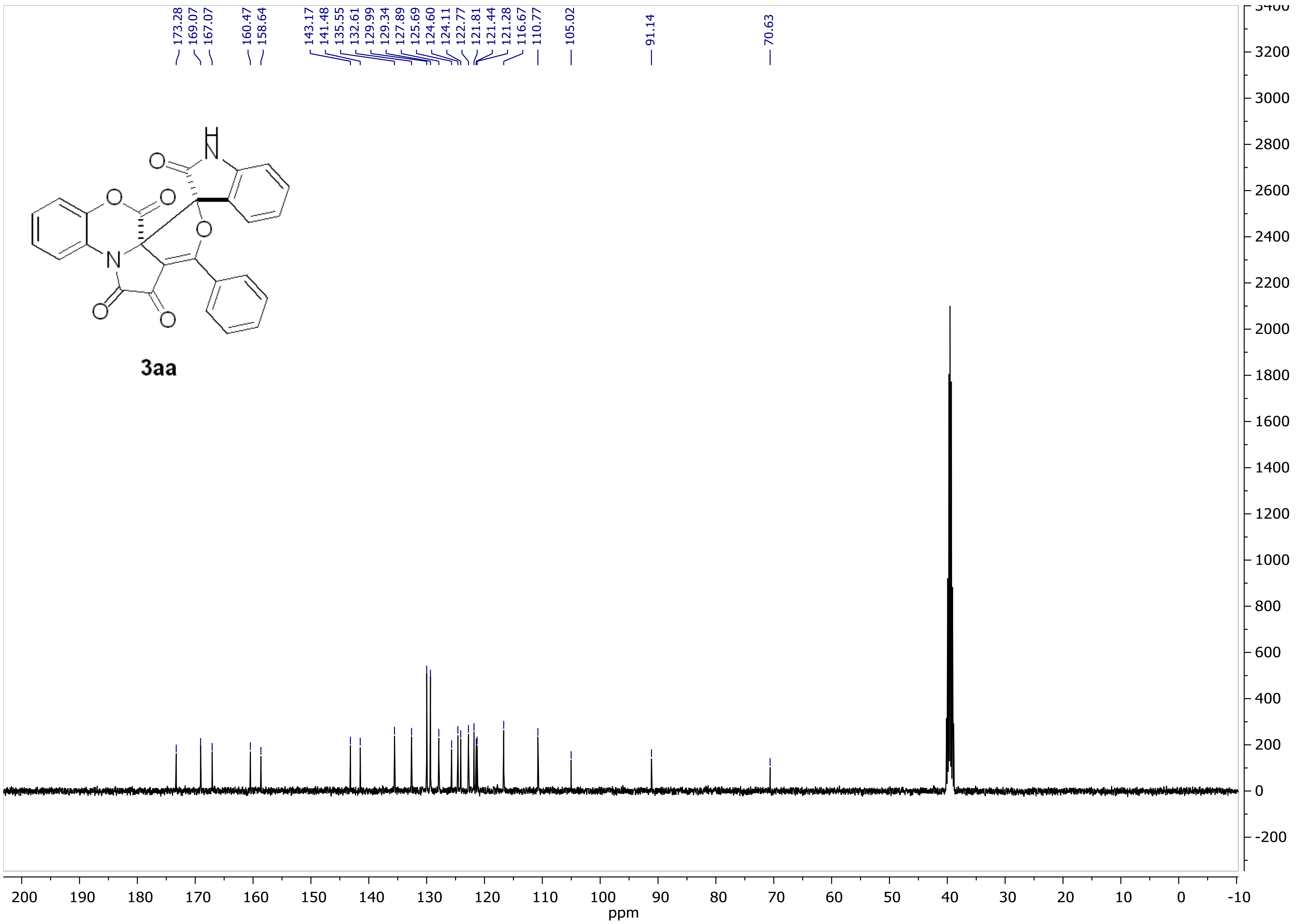

3ab ^{1}H (400 MHz, DMSO-d_6)

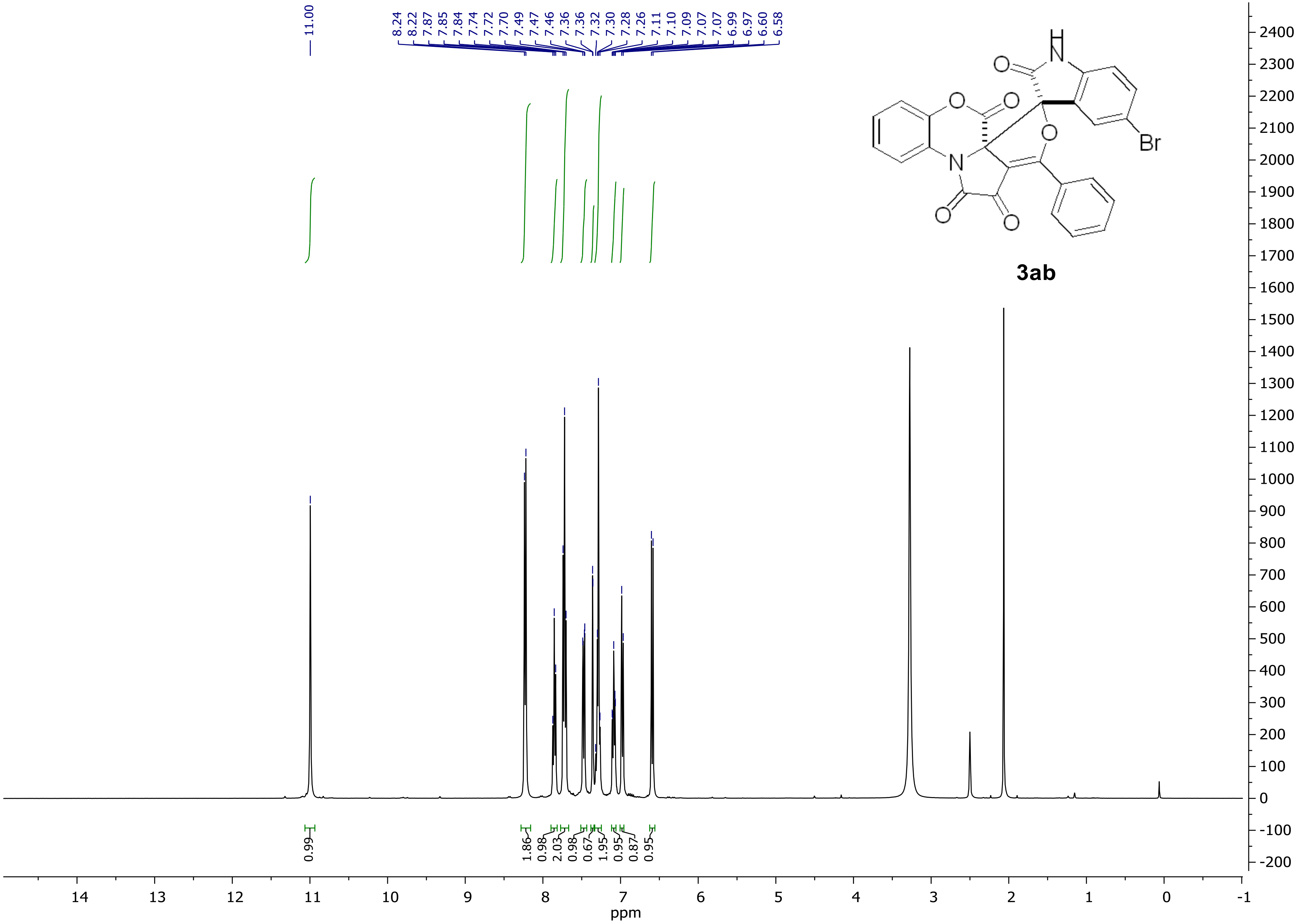

3ab

3ab ^{13}C (101 MHz, DMSO-d_6)

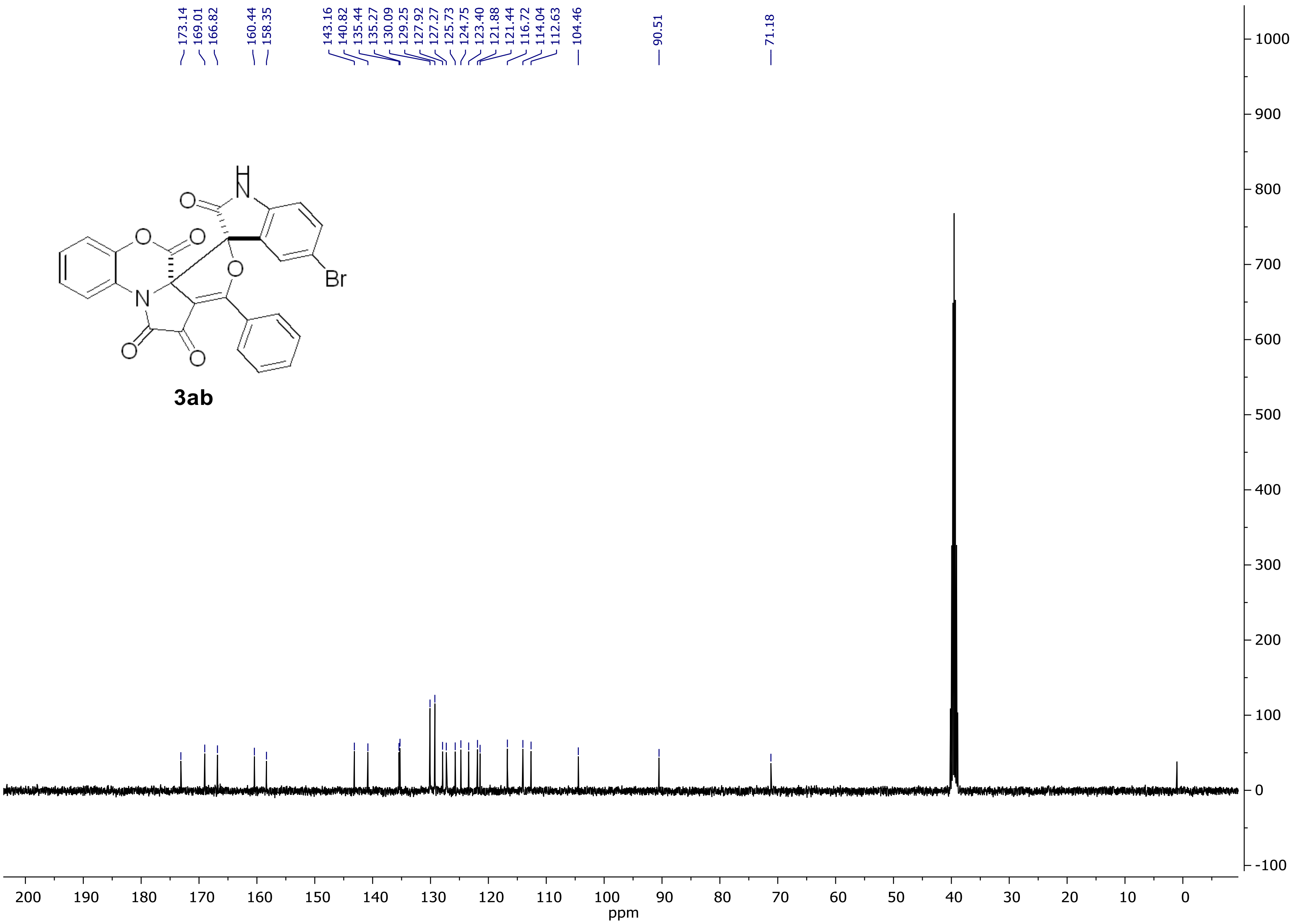

3ac ^{1}H (400 MHz, DMSO-d_6)

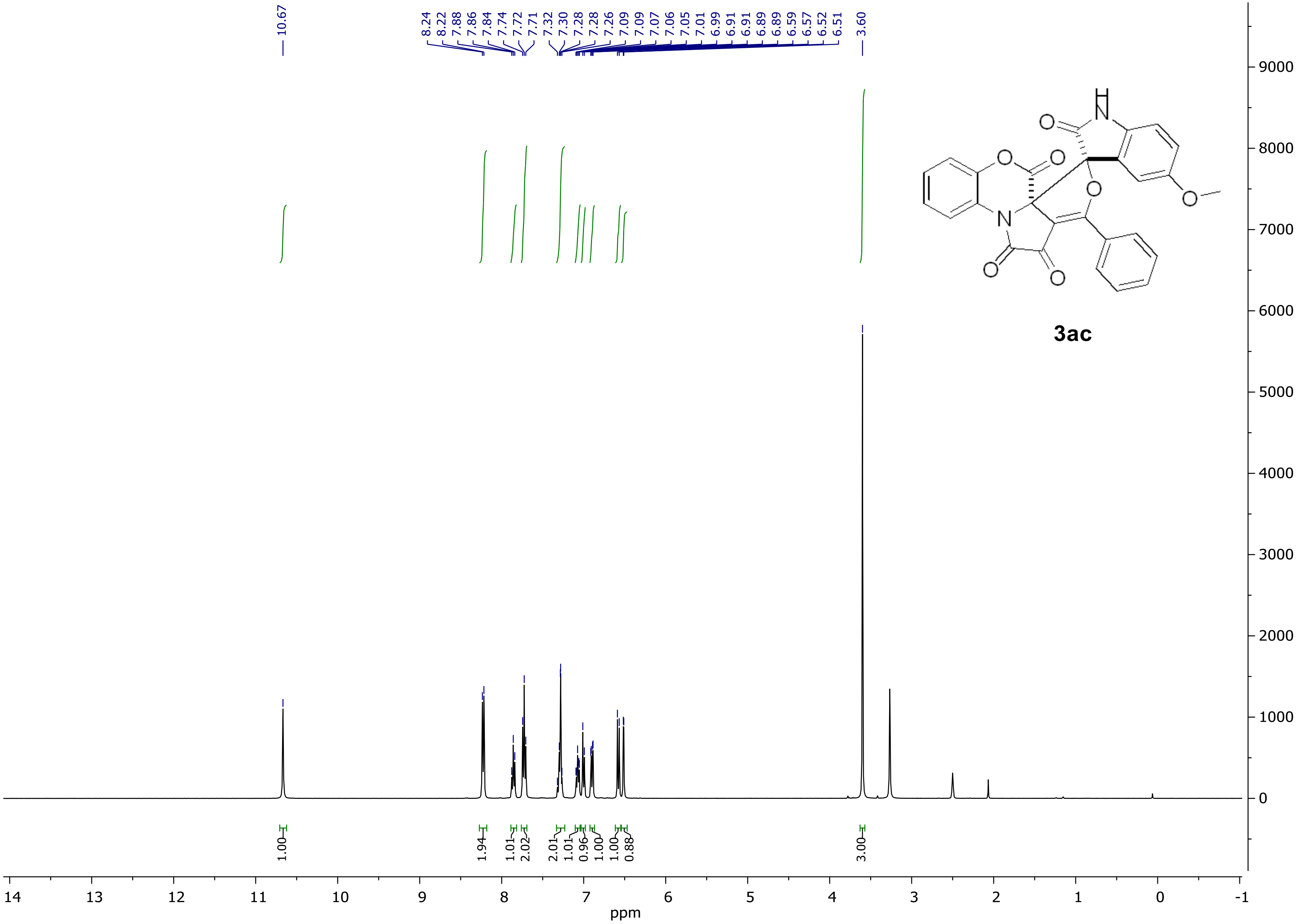

3ac ^{13}C (101 MHz, DMSO-d_6)

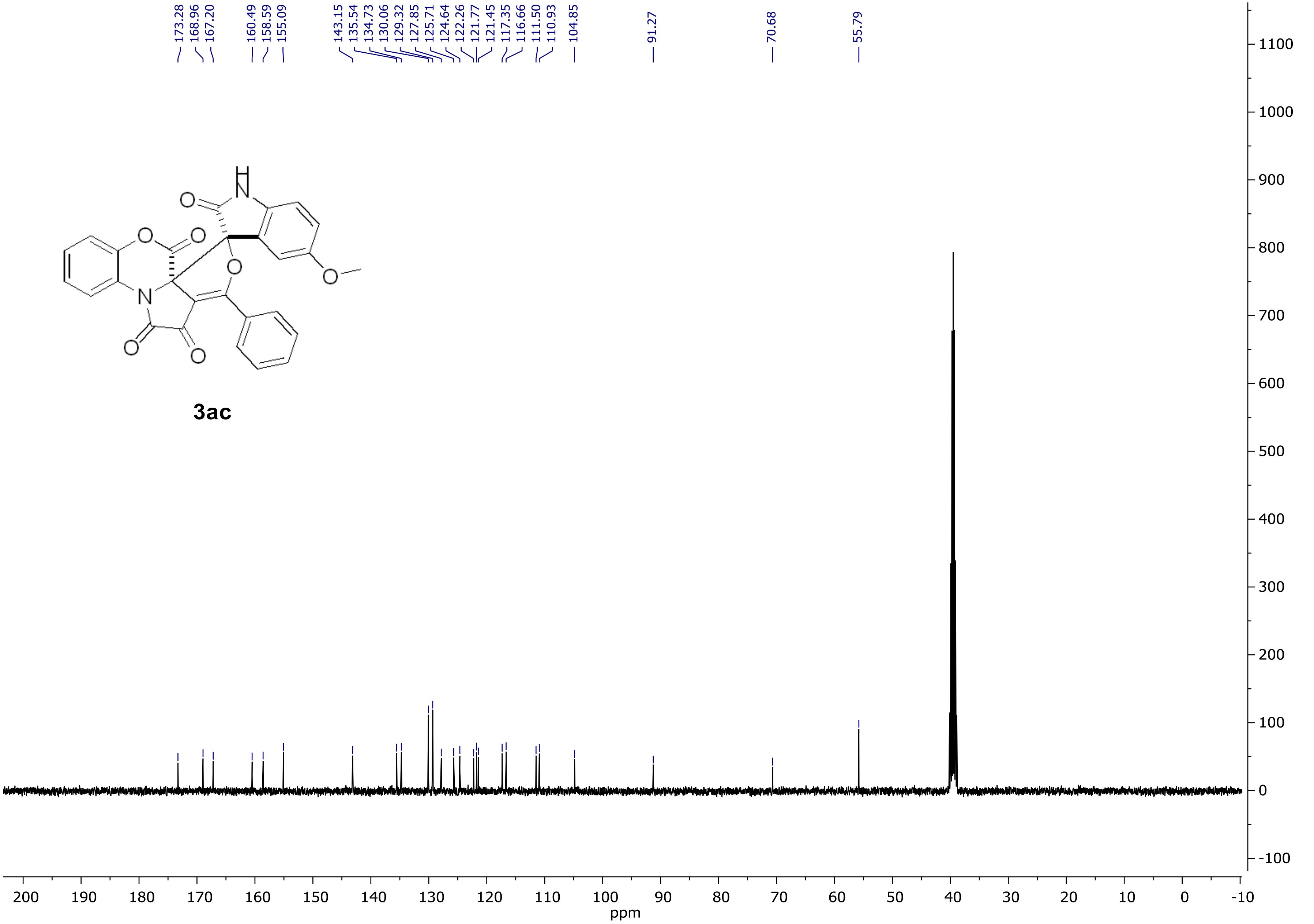

3ad ^{1}H (400 MHz, DMSO-d_6)

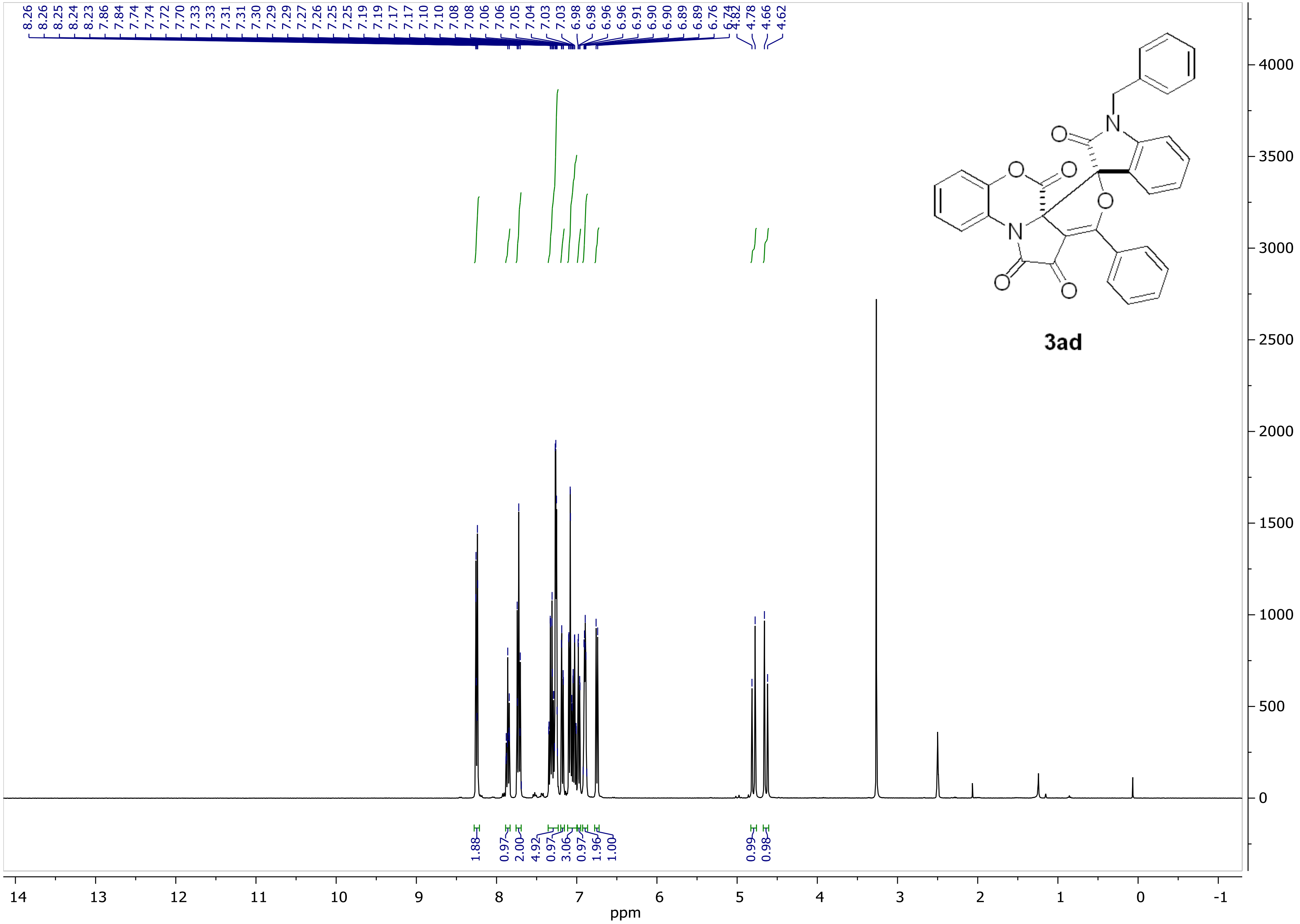

3ad ^{13}C (101 MHz, DMSO-d_6)

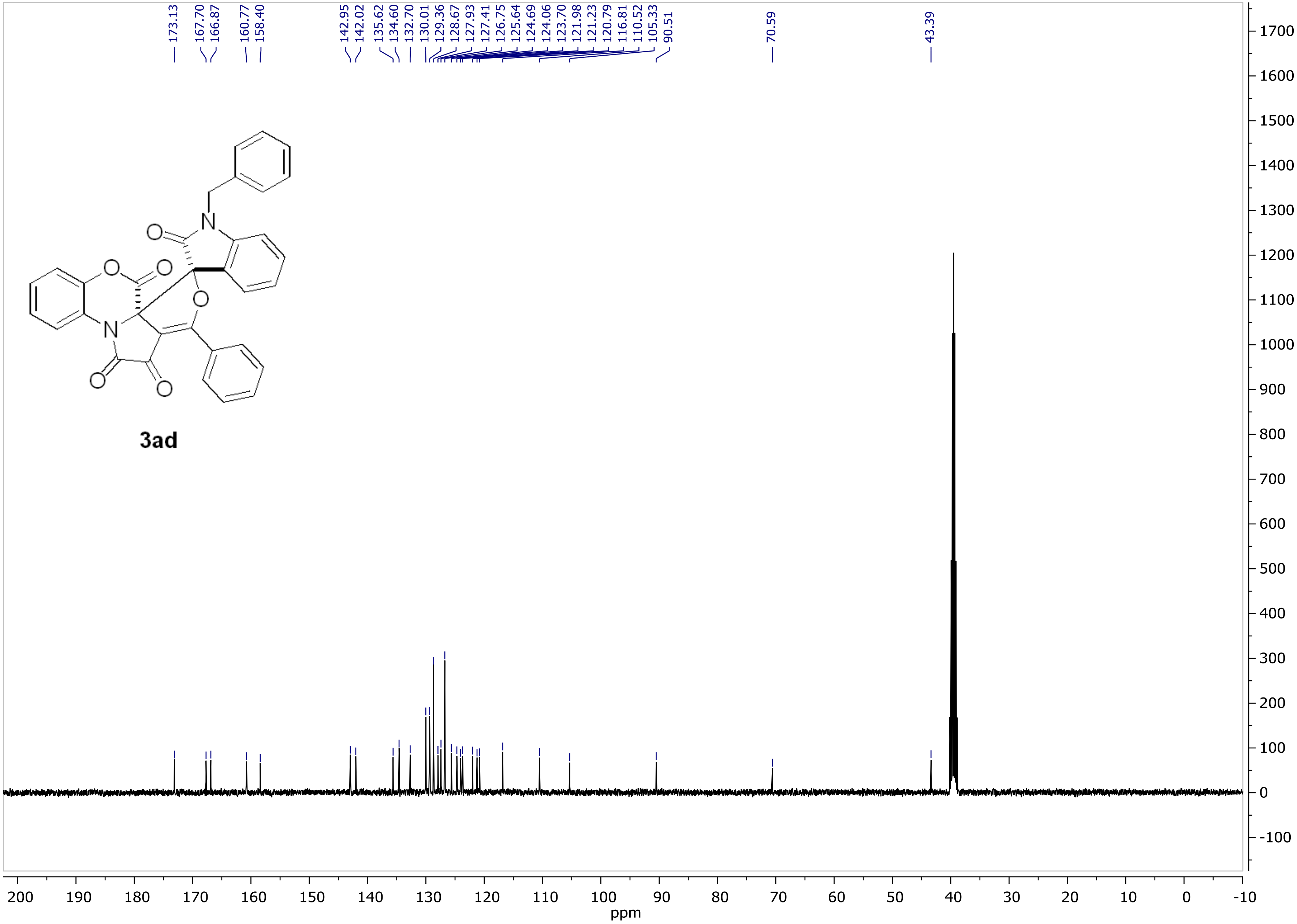

3ad

3ba ^{1}H (400 MHz, DMSO-d_6)

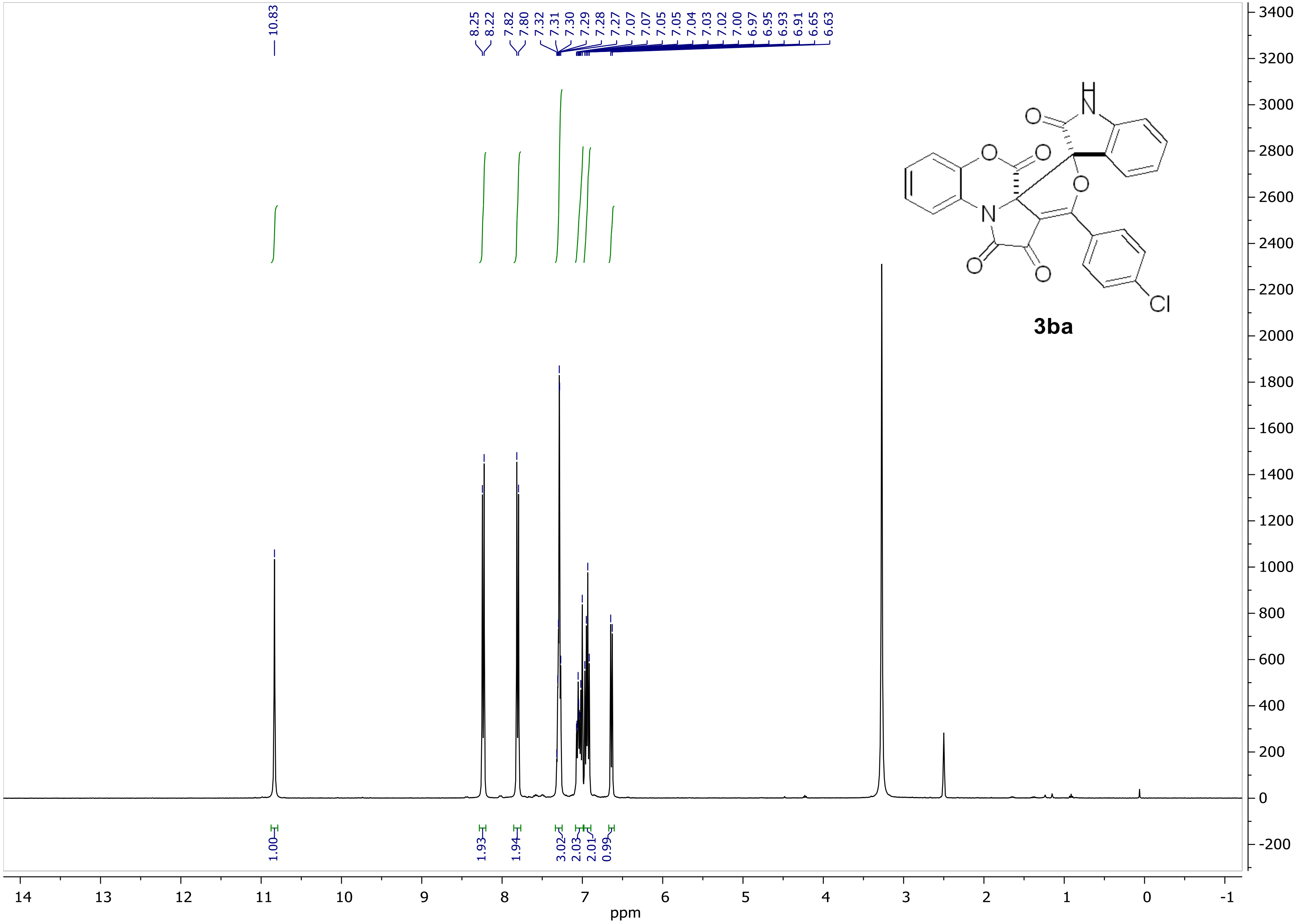

3ba ^{13}C (101 MHz, DMSO-d_6)

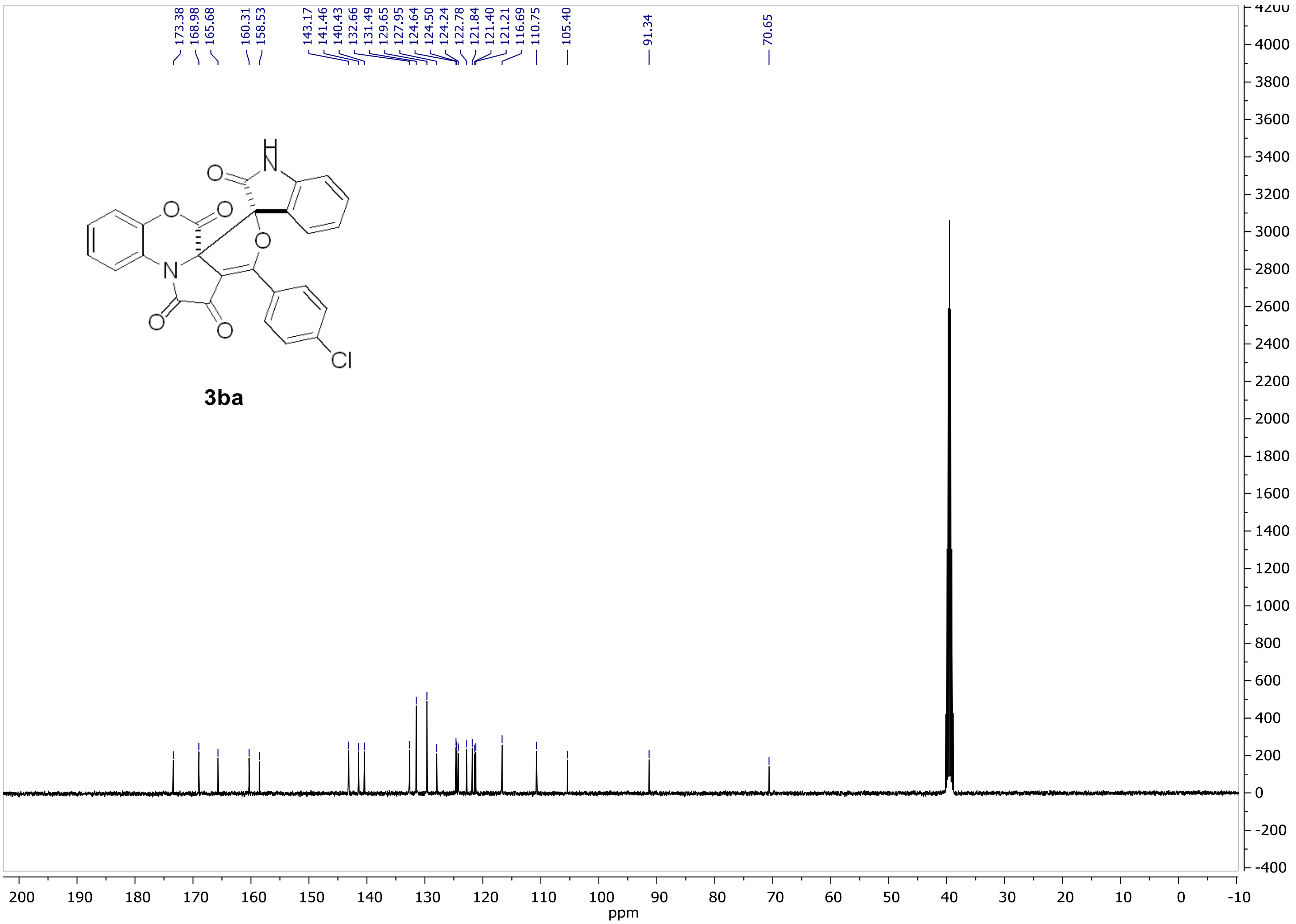

3ca ^{1}H (400 MHz, DMSO-d_6)

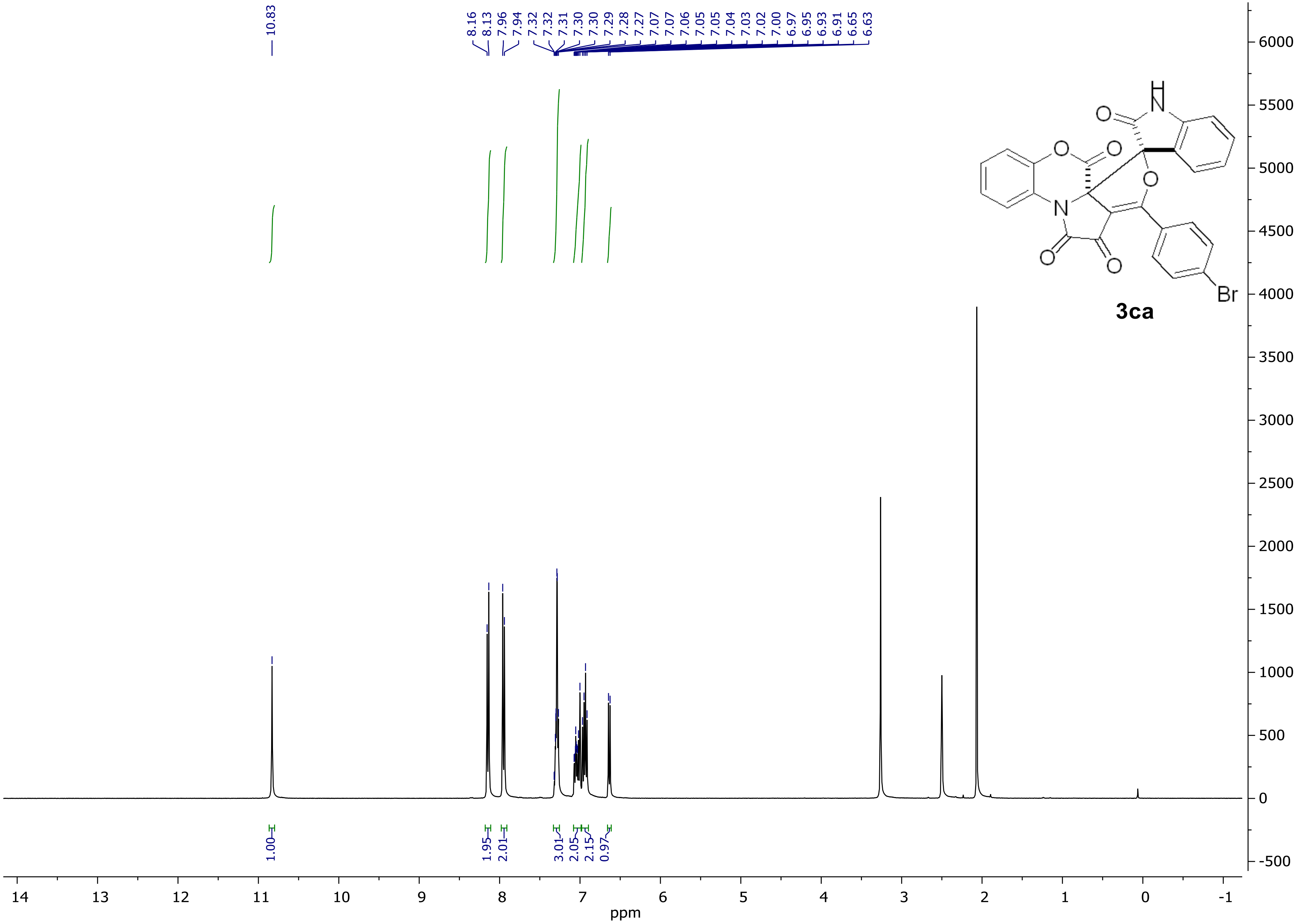

3ca ^{13}C (101 MHz, DMSO-d_6)

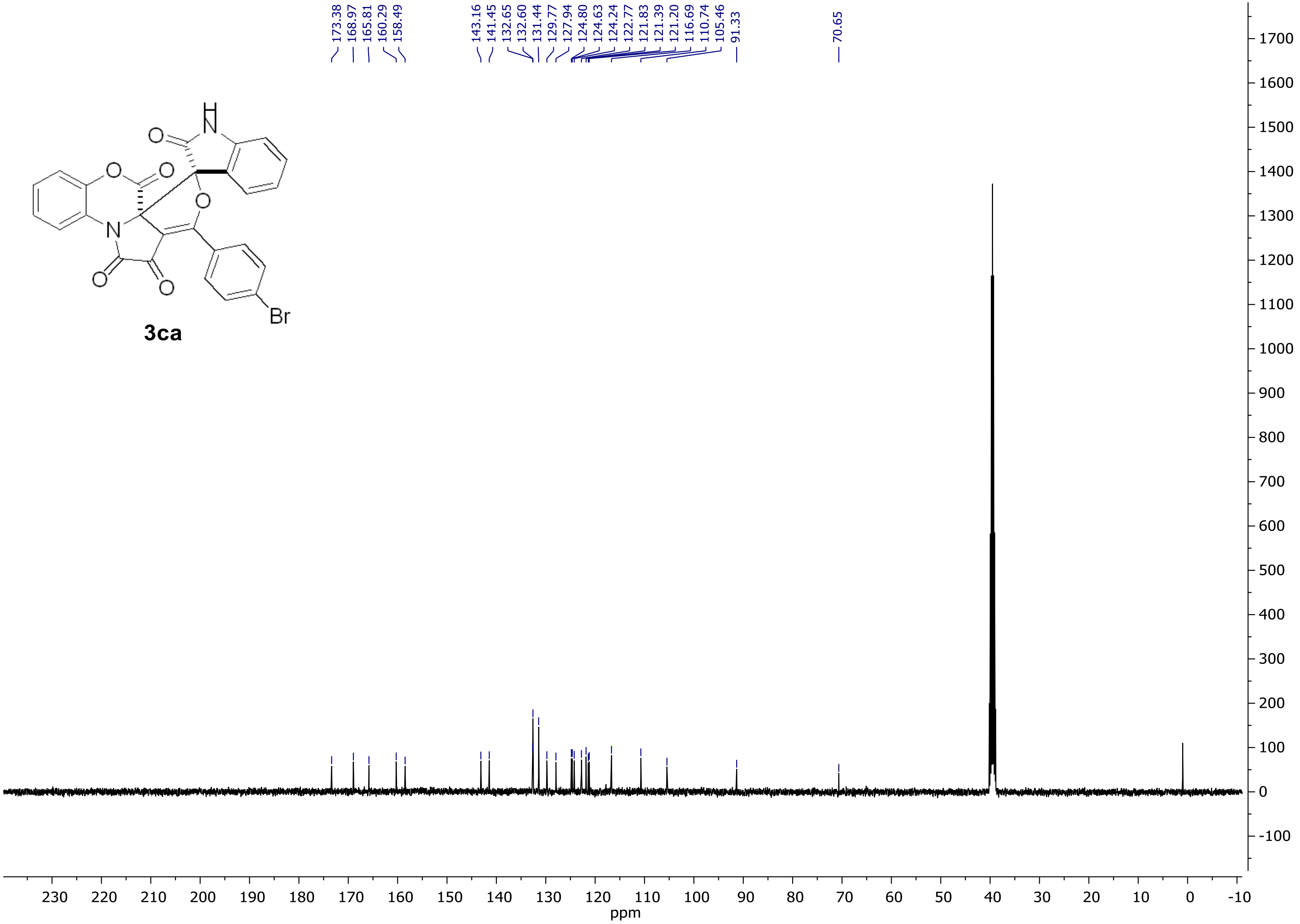

3da ^{1}H (400 MHz, DMSO-d_6)

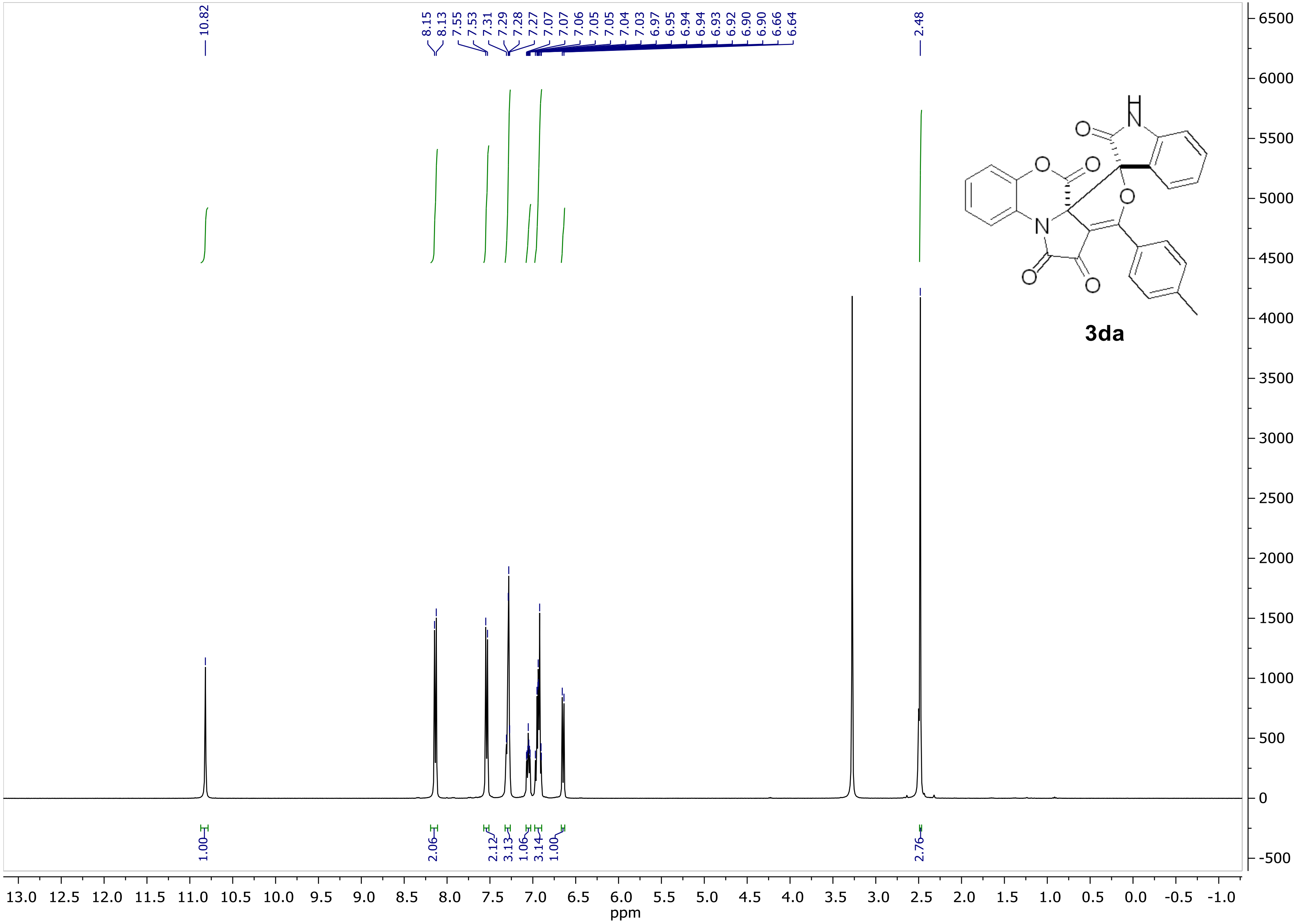

3da ^{13}C (101 MHz, DMSO-d_6)

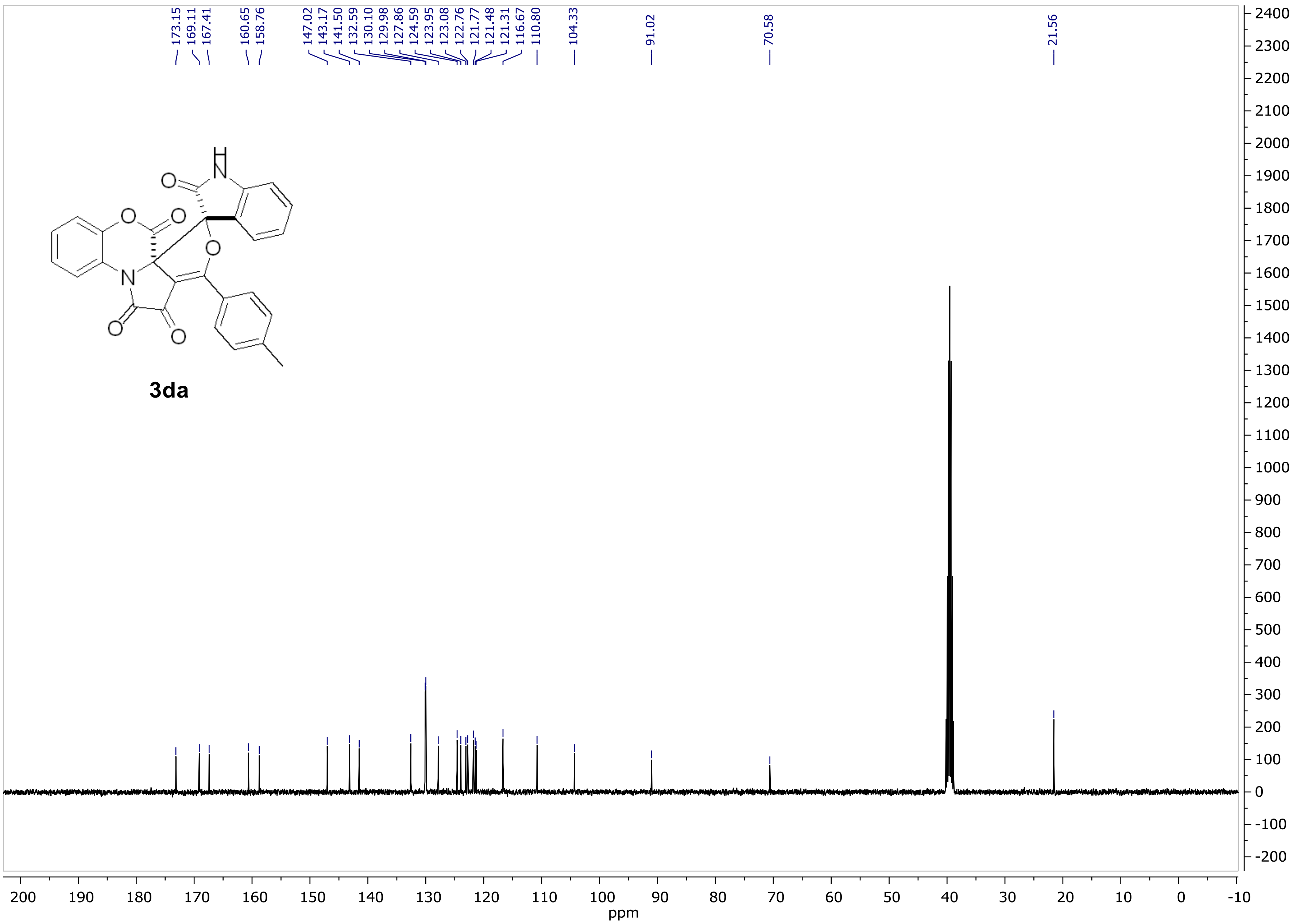

3ea ^{1}H (400 MHz, DMSO-d_6)

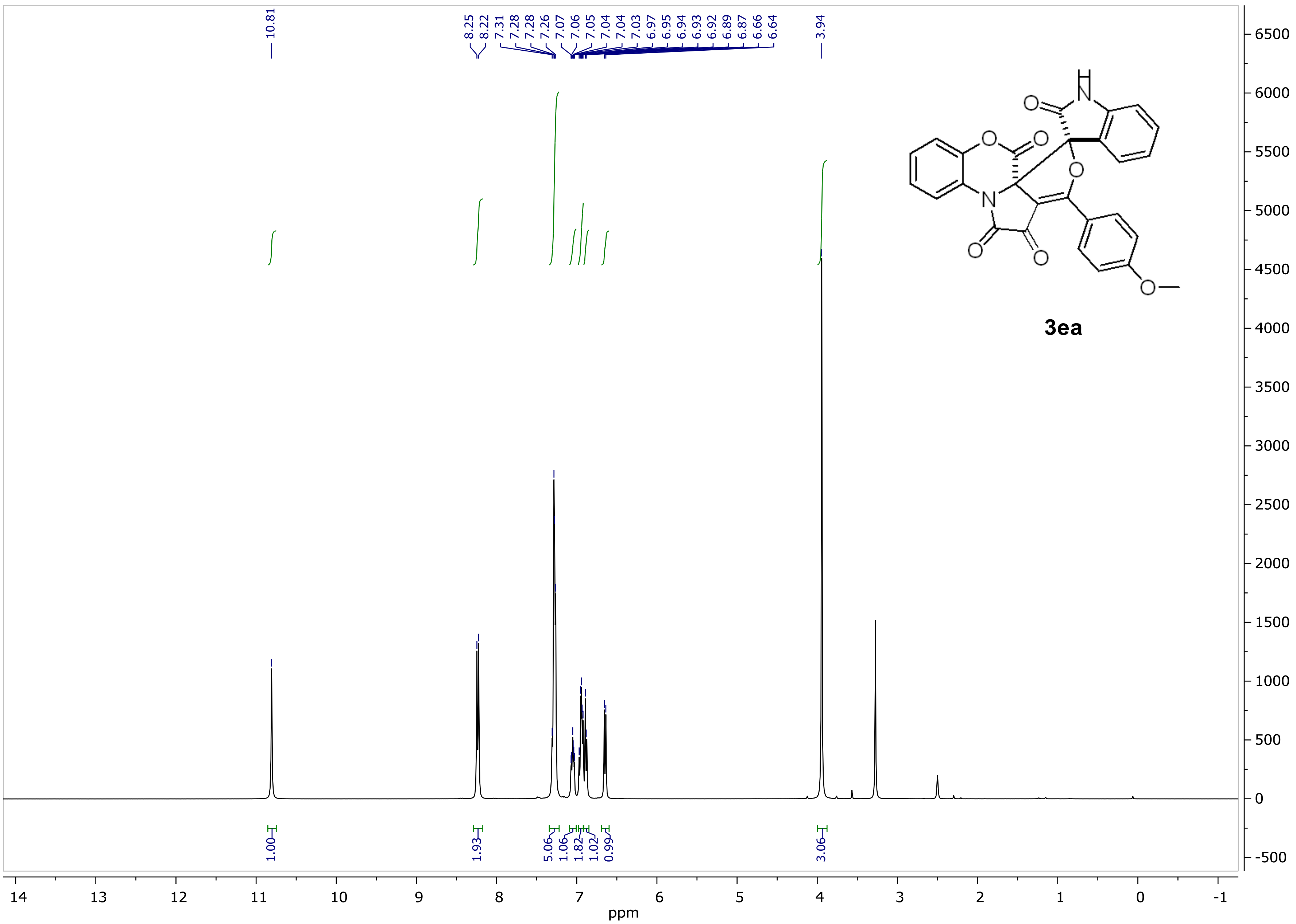

3ea ^{13}C (101 MHz, DMSO-d_6)

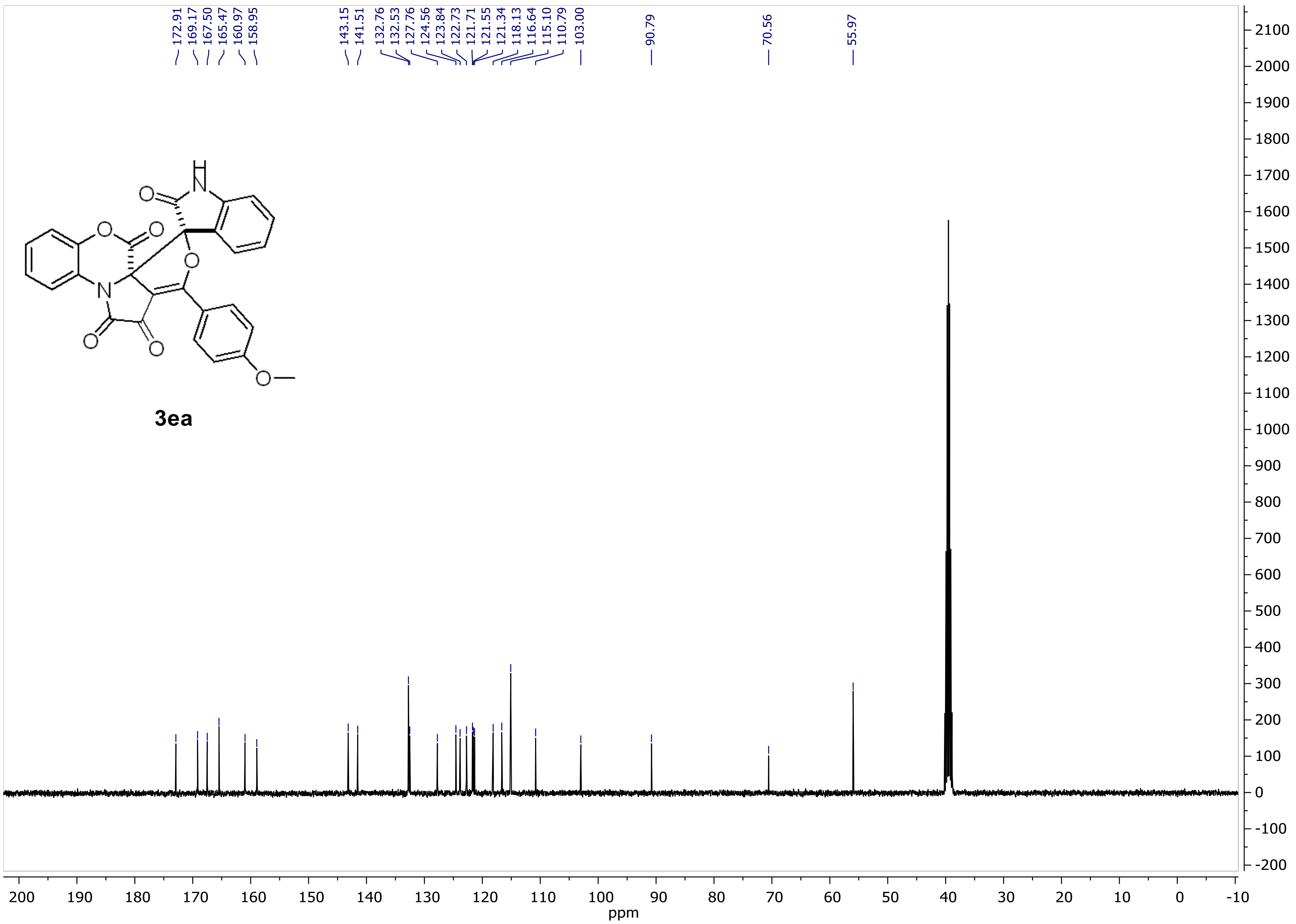

3fa ^{1}H (400 MHz, DMSO-d_6)

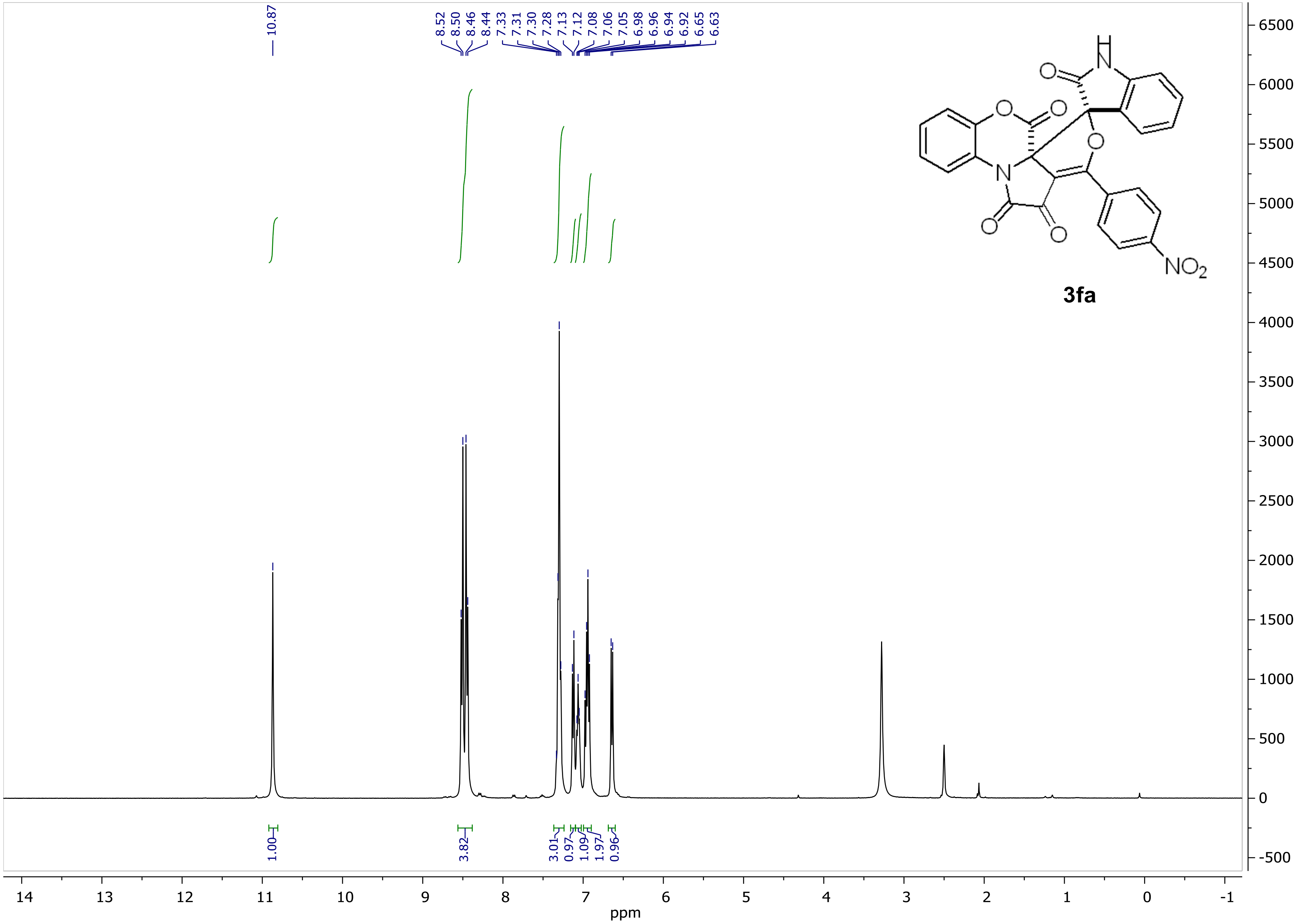

3fa ^{13}C (101 MHz, DMSO-d_6)

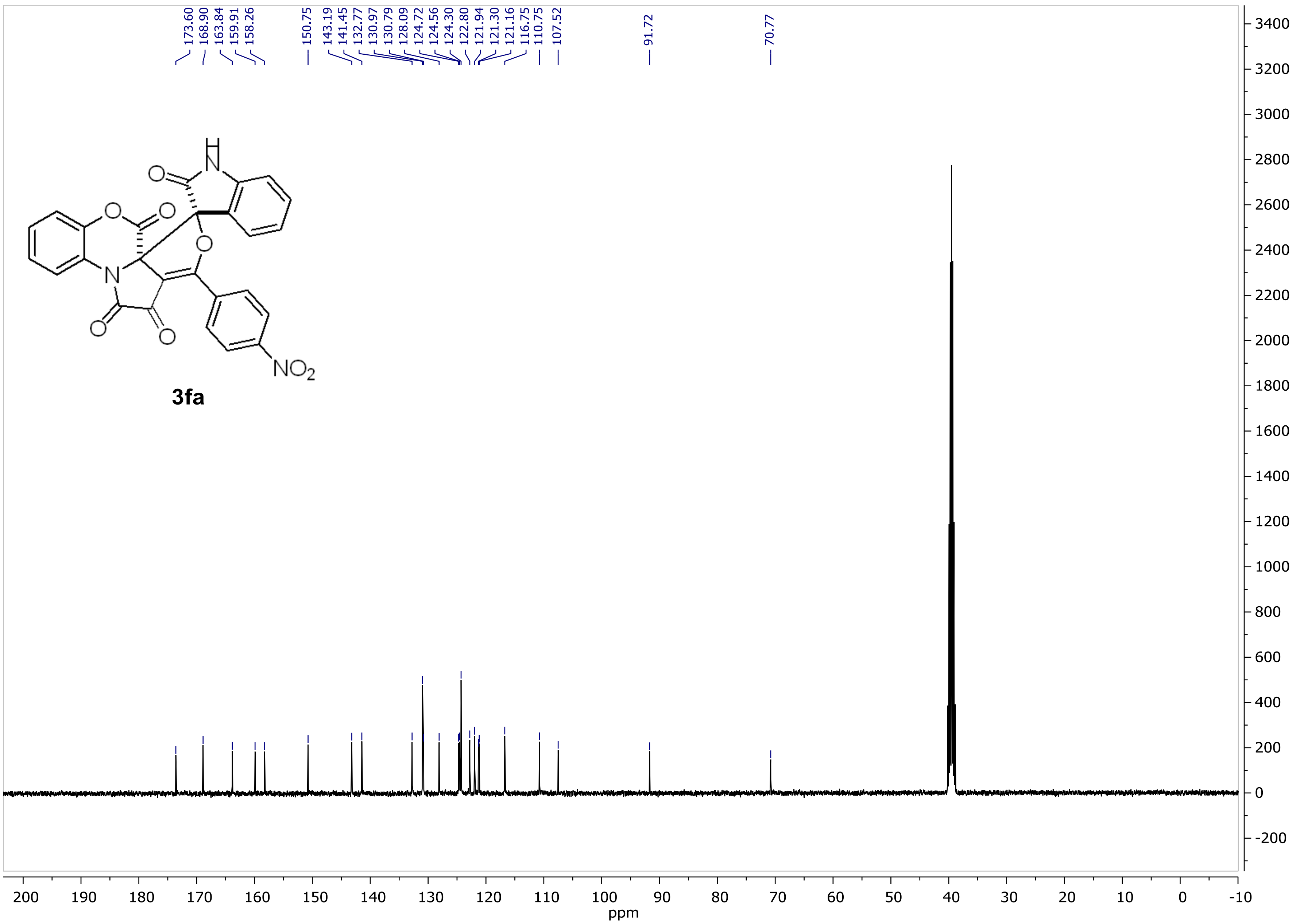

3ga ^{1}H (400 MHz, DMSO-d_6)

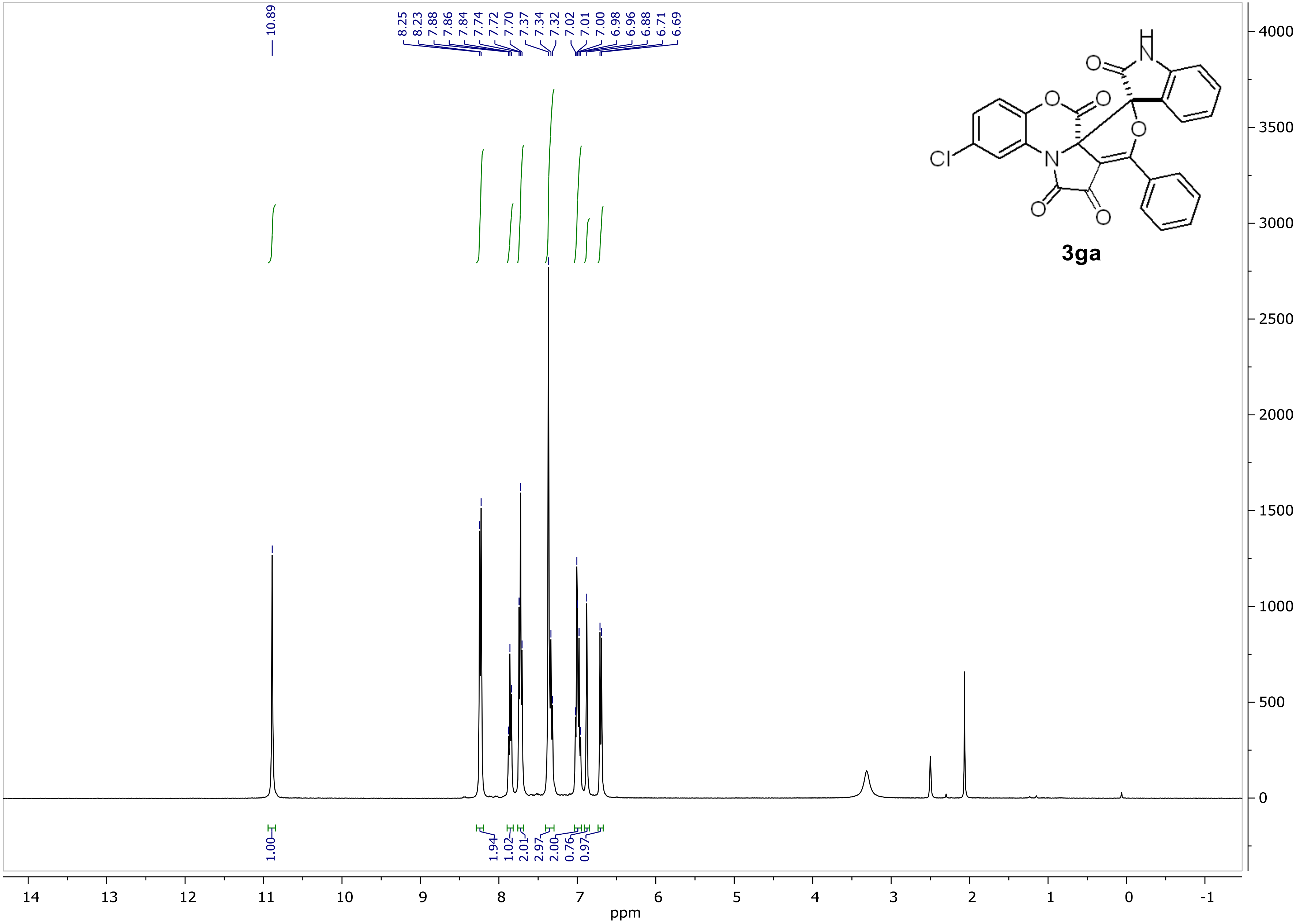

3ga ^{13}C (101 MHz, DMSO-d_6)

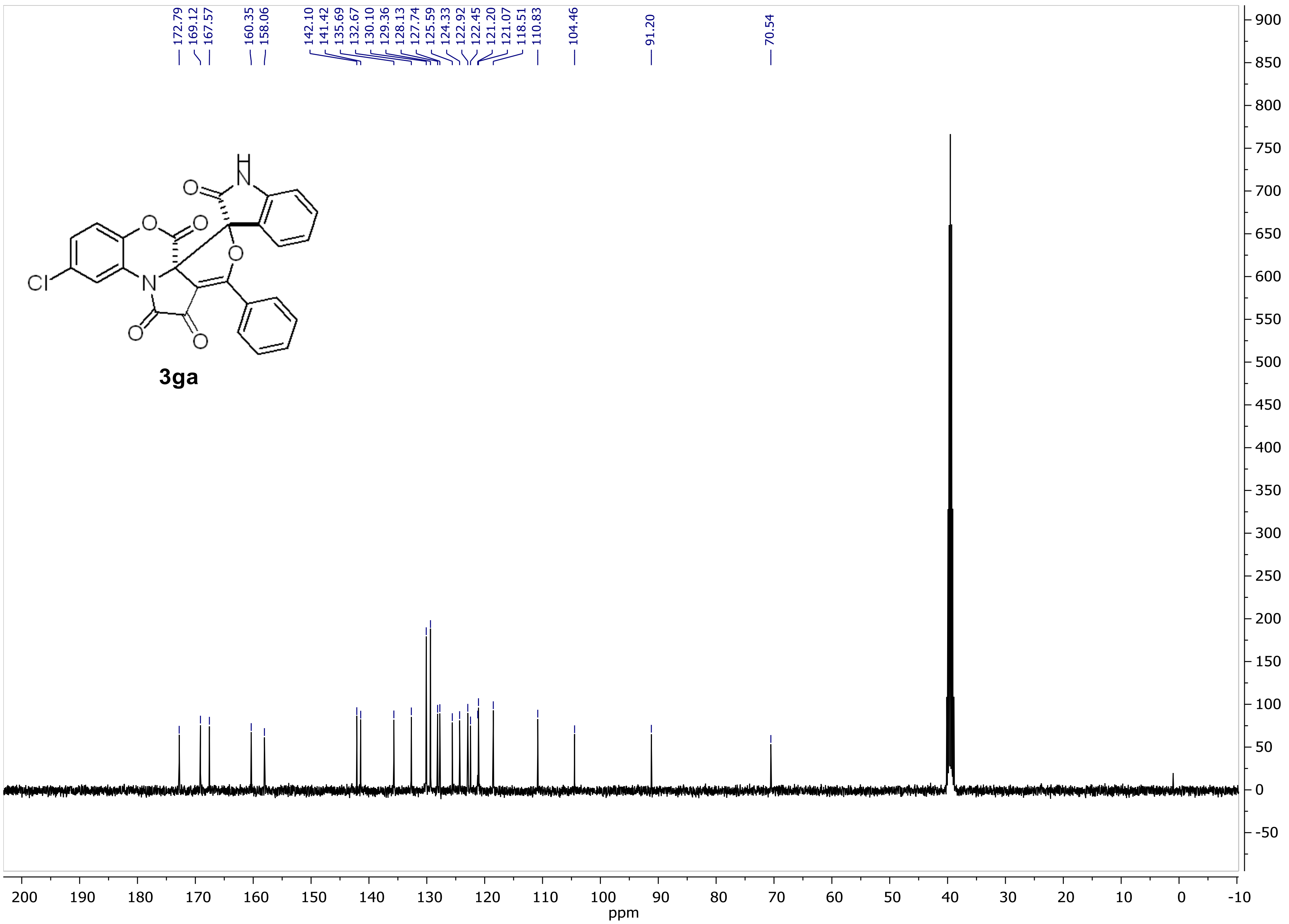

3ha ¹H (400 MHz, DMSO-d_6) ACN solvate 1:1

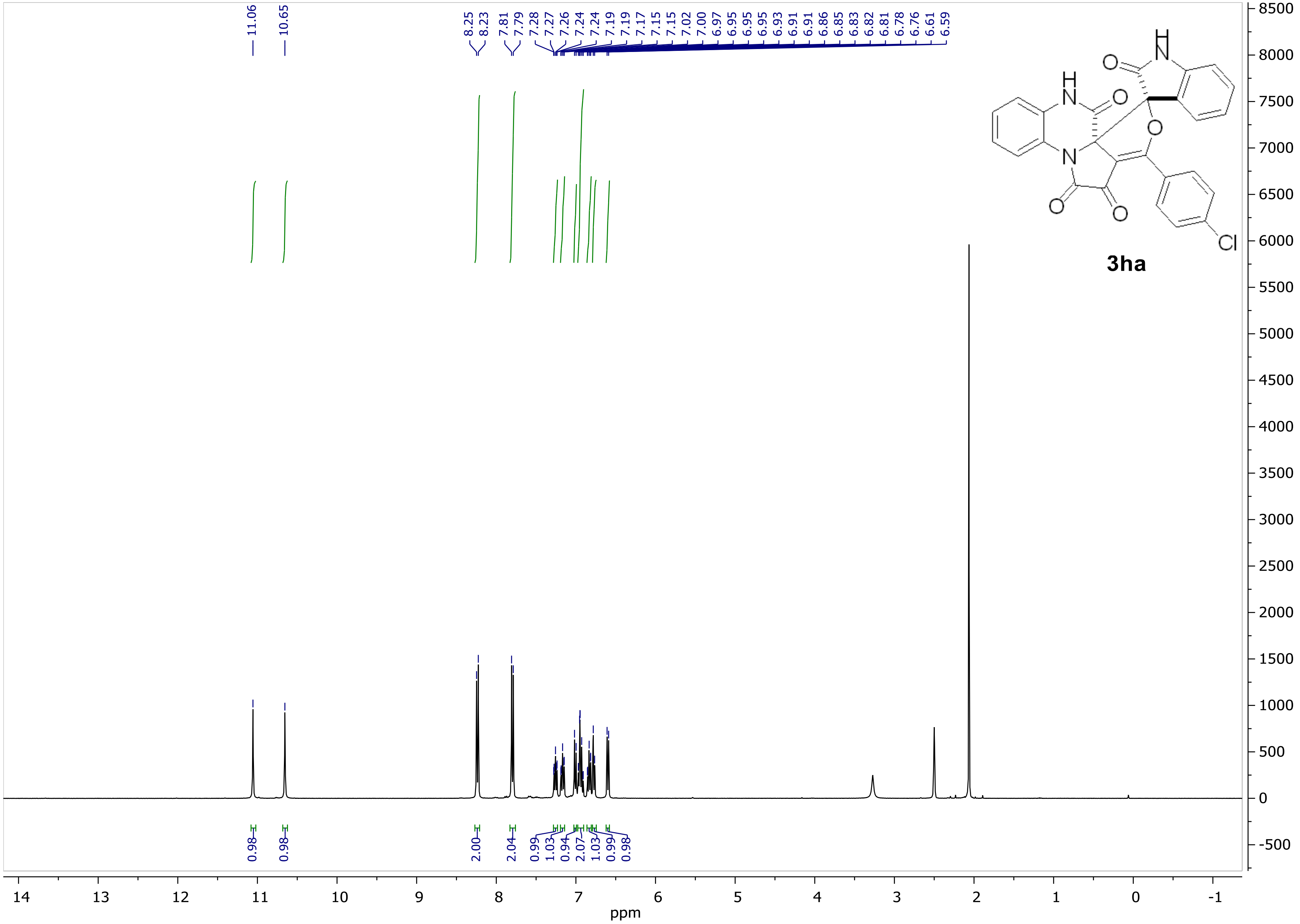

3ha ^{13}C (101 MHz, DMSO-d_6) ACN solvate 1:1

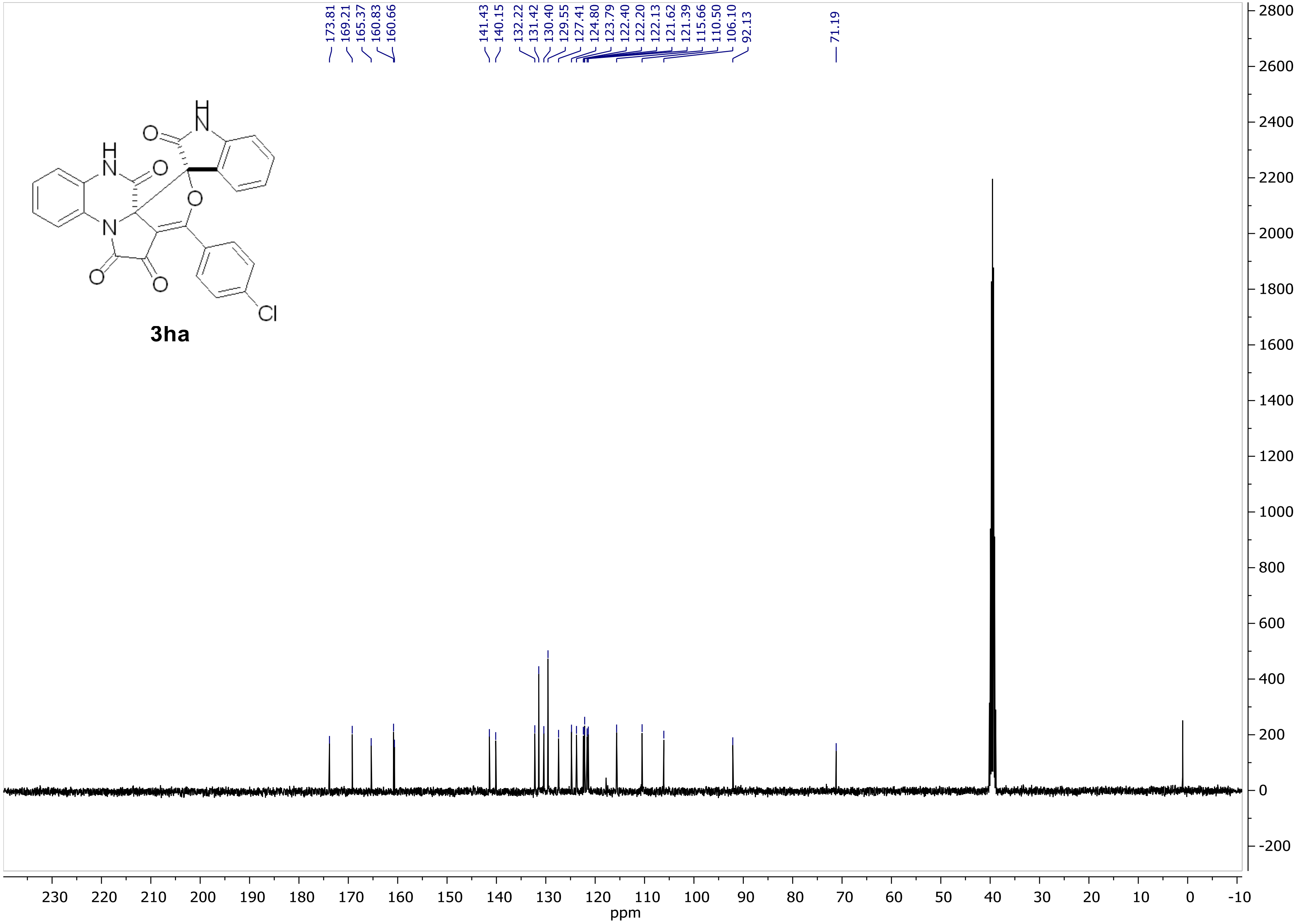

3ha

3ia ^{1}H (400 MHz, DMSO-d_6)

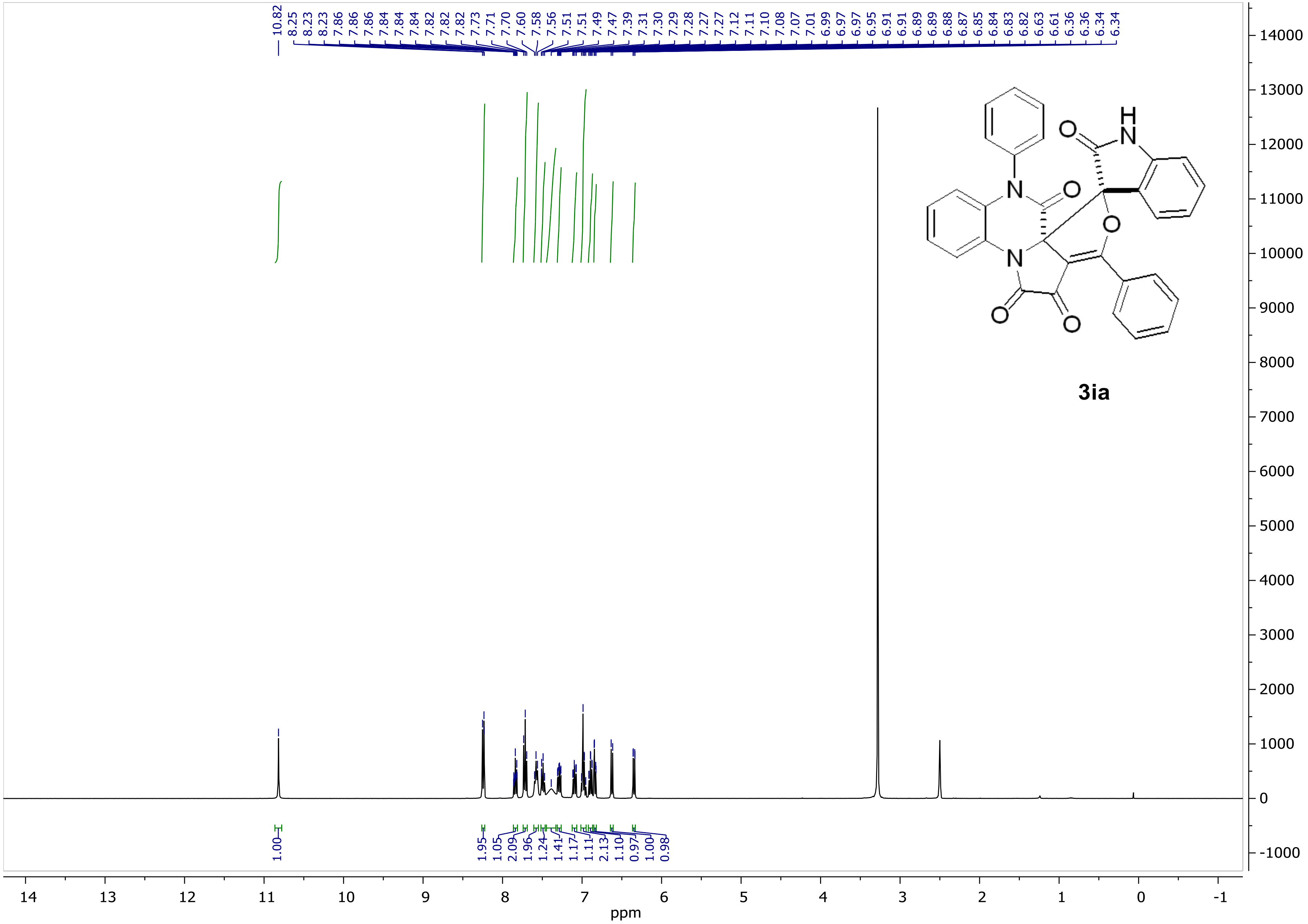

3ia ^{13}C (101 MHz, DMSO-d_6)

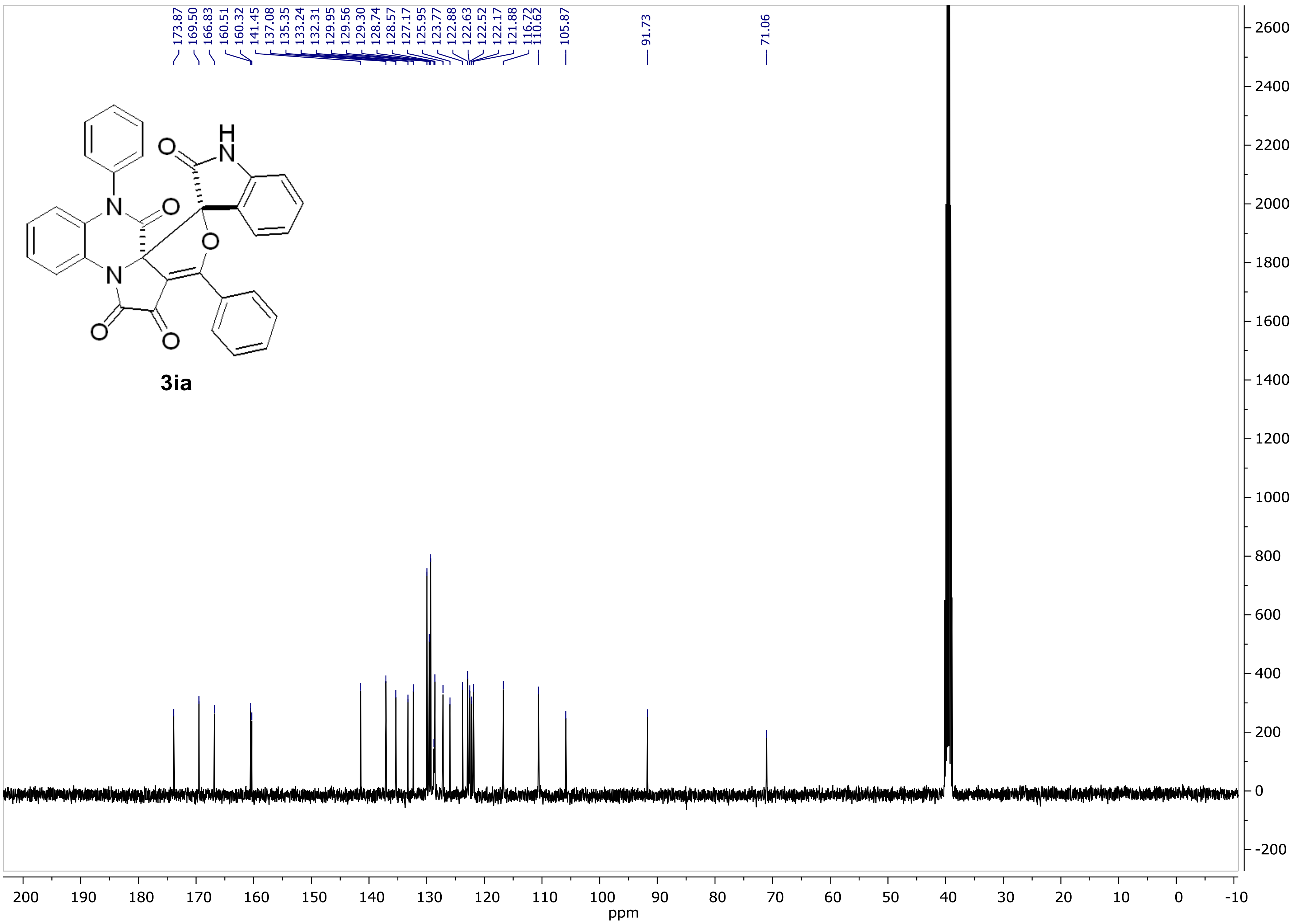

3ia (control experiment) ^{1}H (400 MHz, DMSO-d_6)

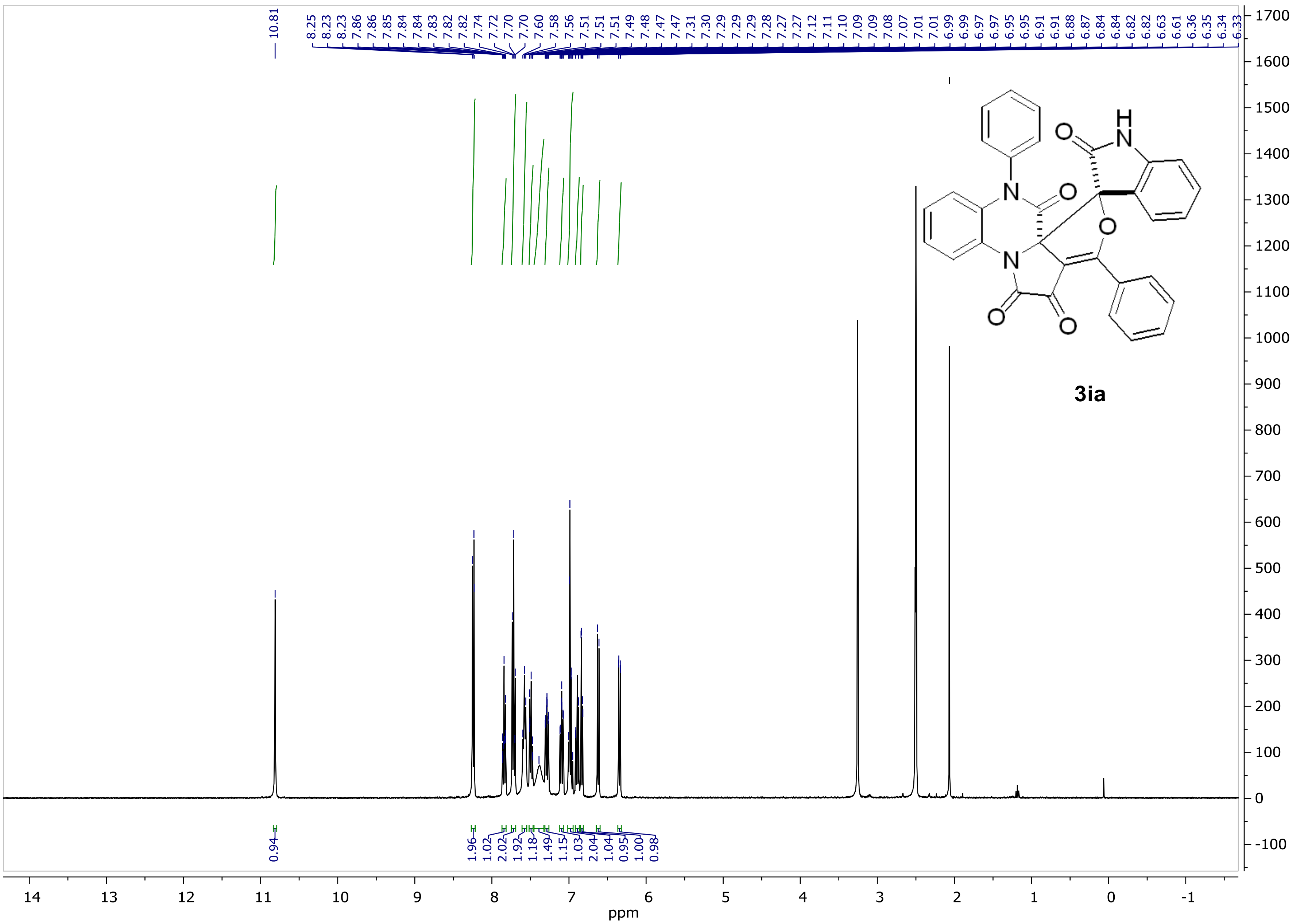

3ia (control experiment) ^{13}C (101 MHz, DMSO-d_6)

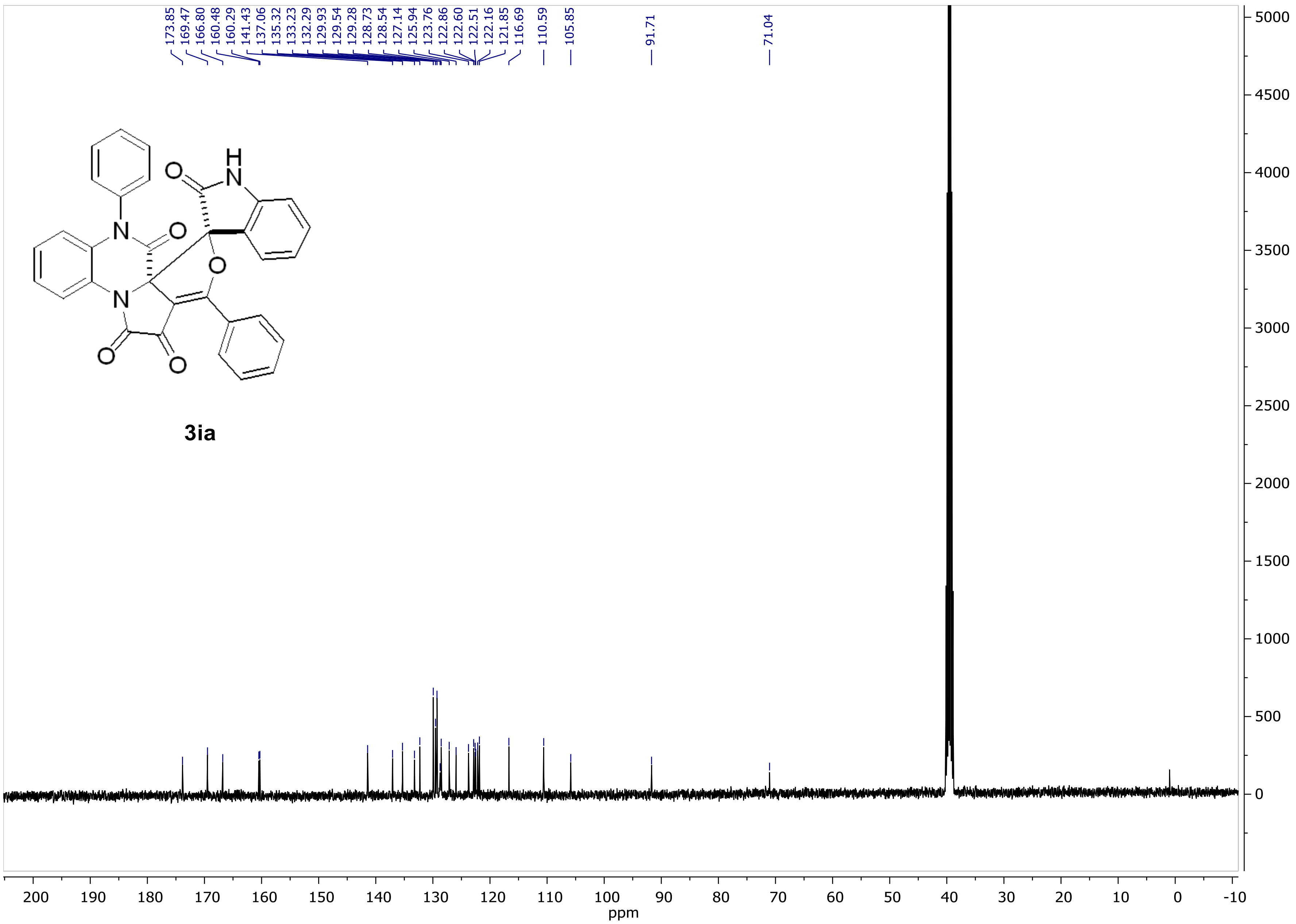

3ja ^{1}H (400 MHz, DMSO-d_6) 1,4-dioxane solvate 1:1

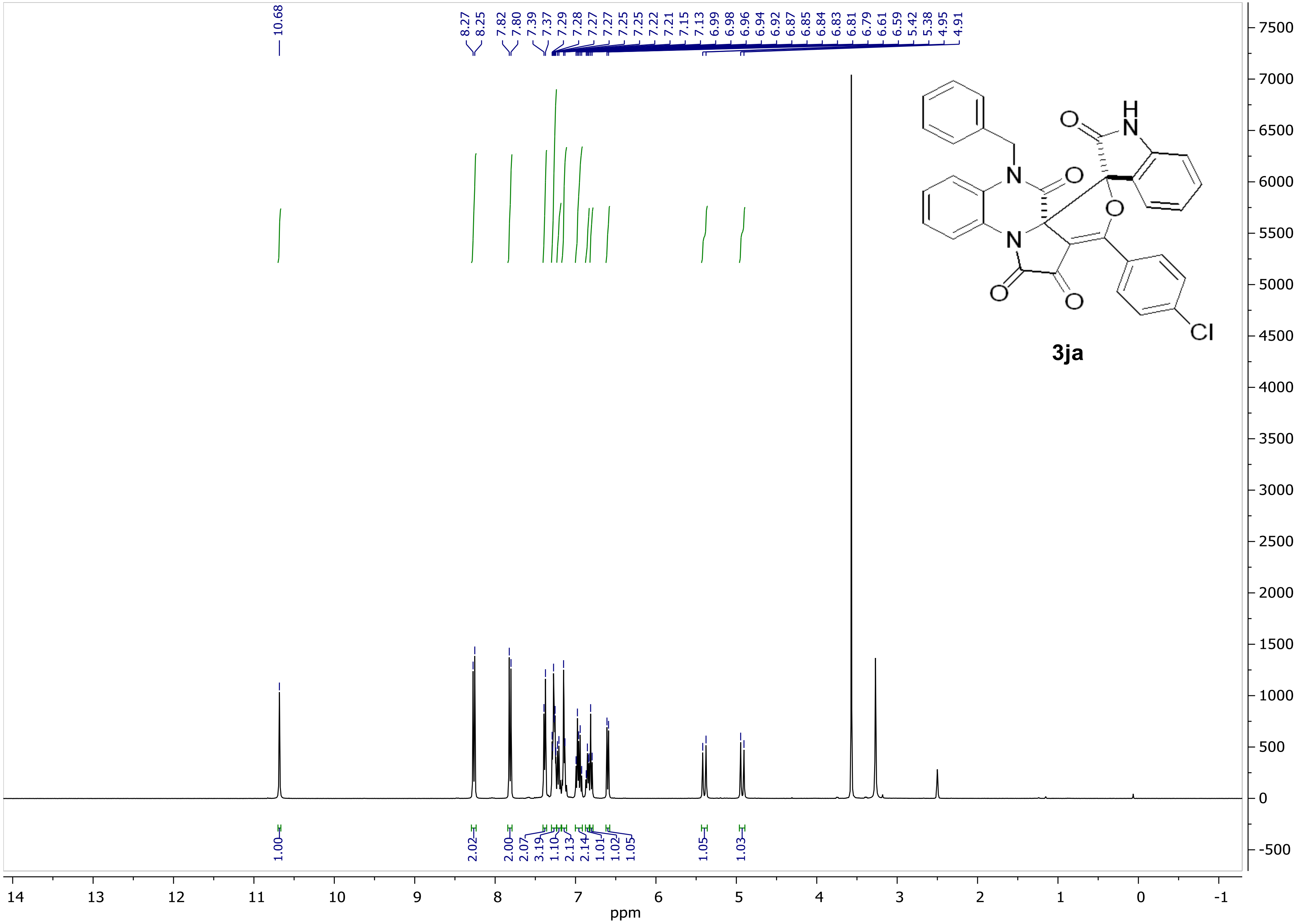

3ja ^{13}C (101 MHz, DMSO-d_6) 1,4-dioxane solvate 1:1

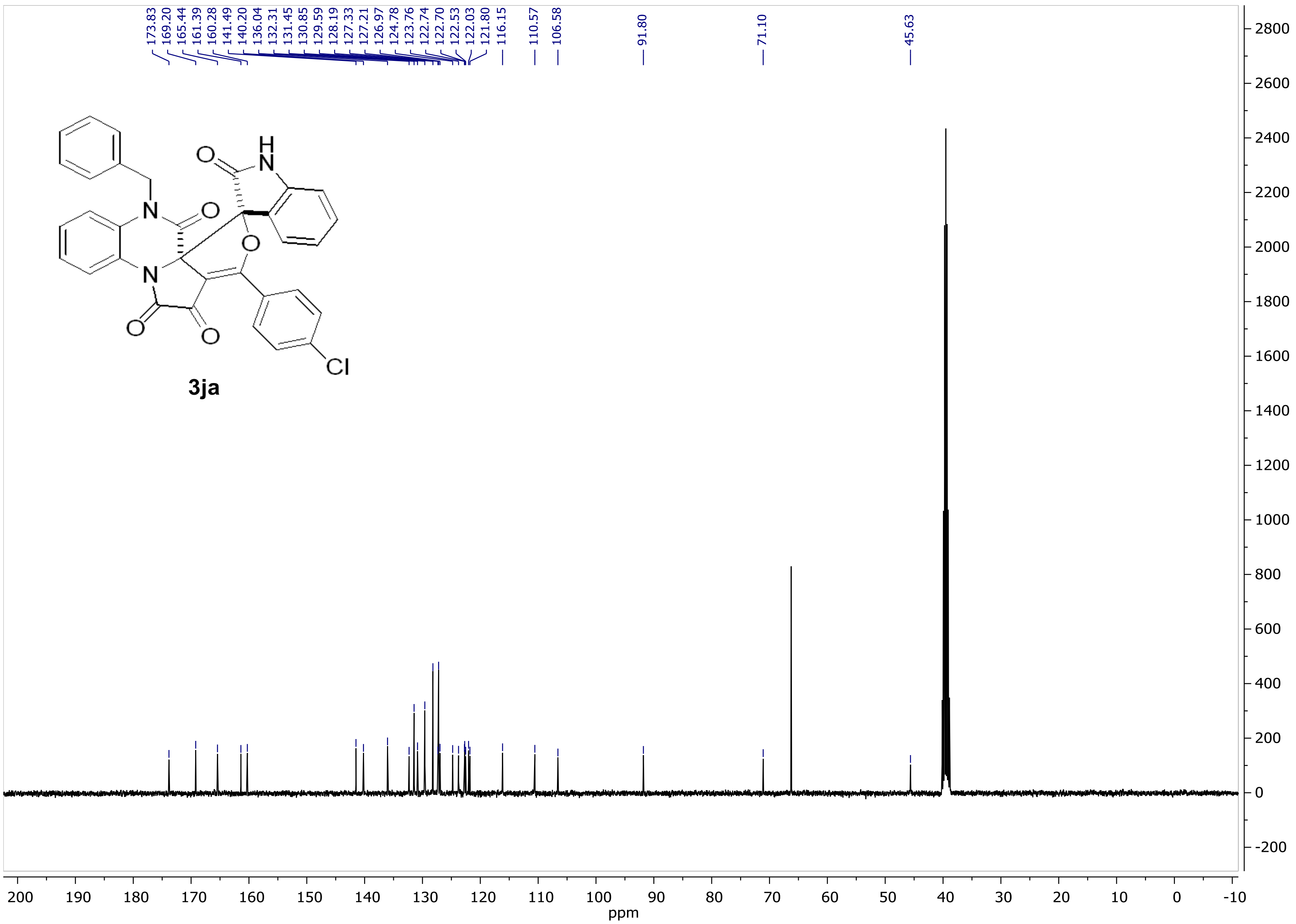

3ka ^{1}H (400 MHz, DMSO-d_6)

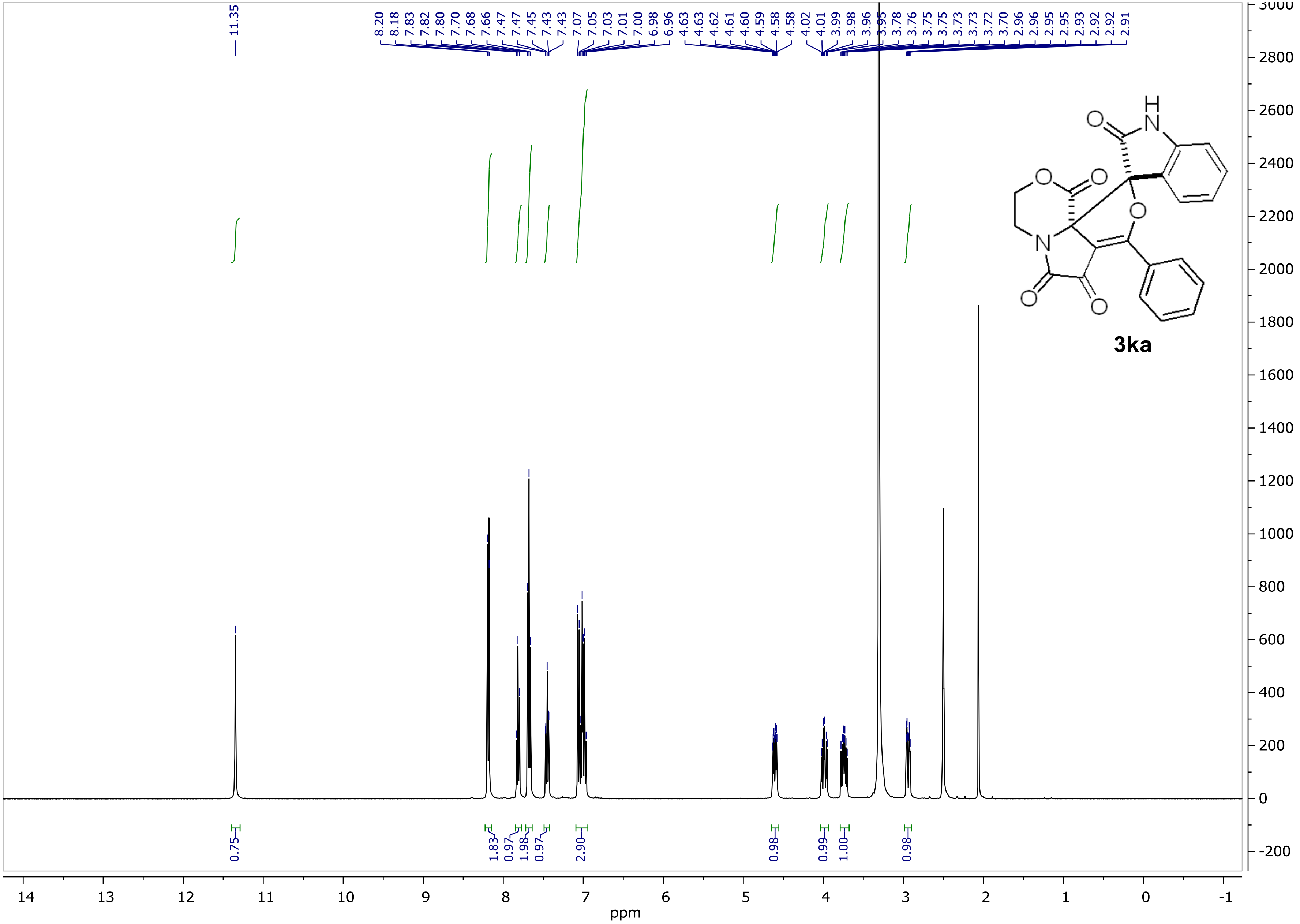

3ka ^{13}C (101 MHz, DMSO-d_6)

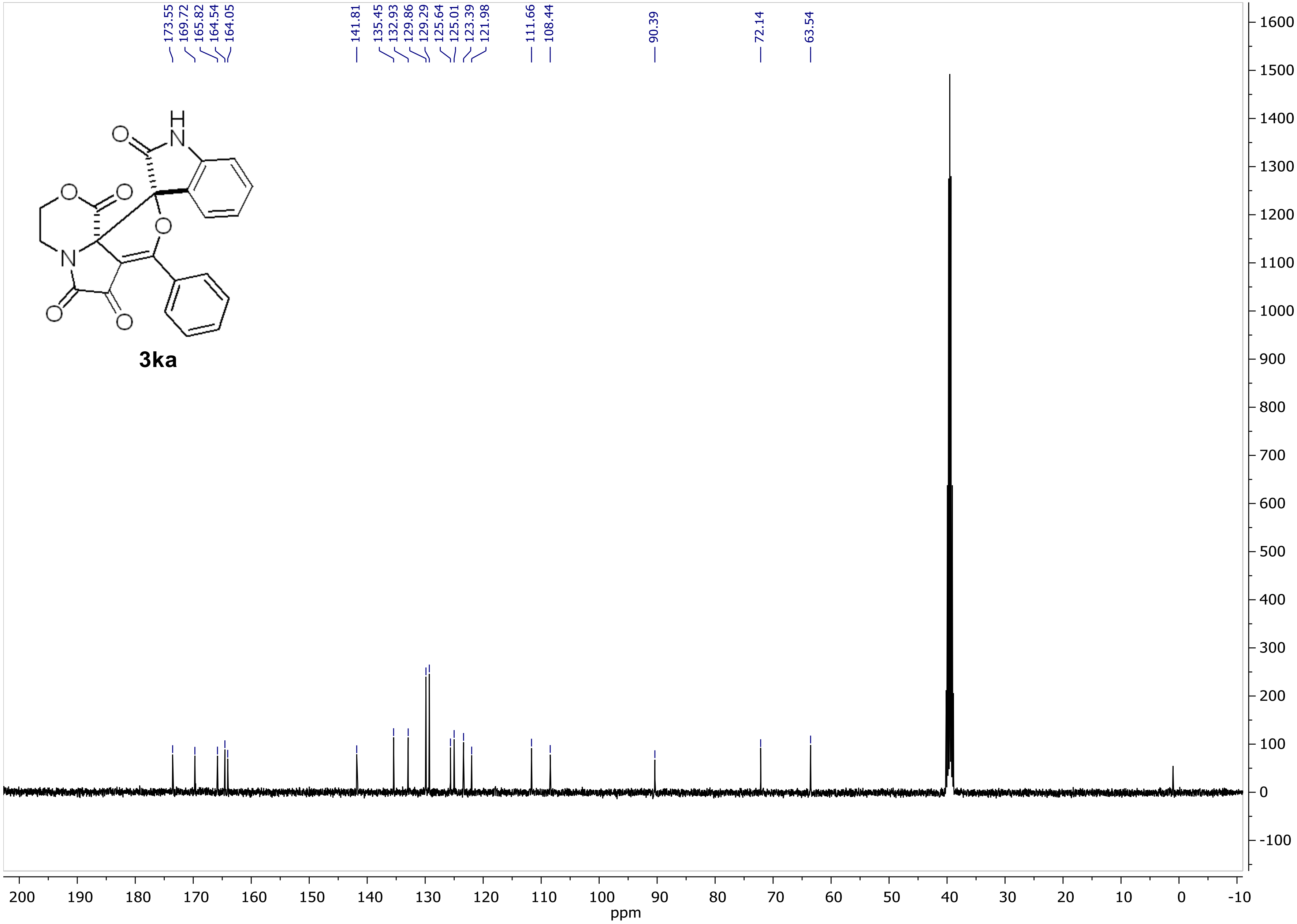

NMR charts of compound 3aa after dissolution and 24 h later

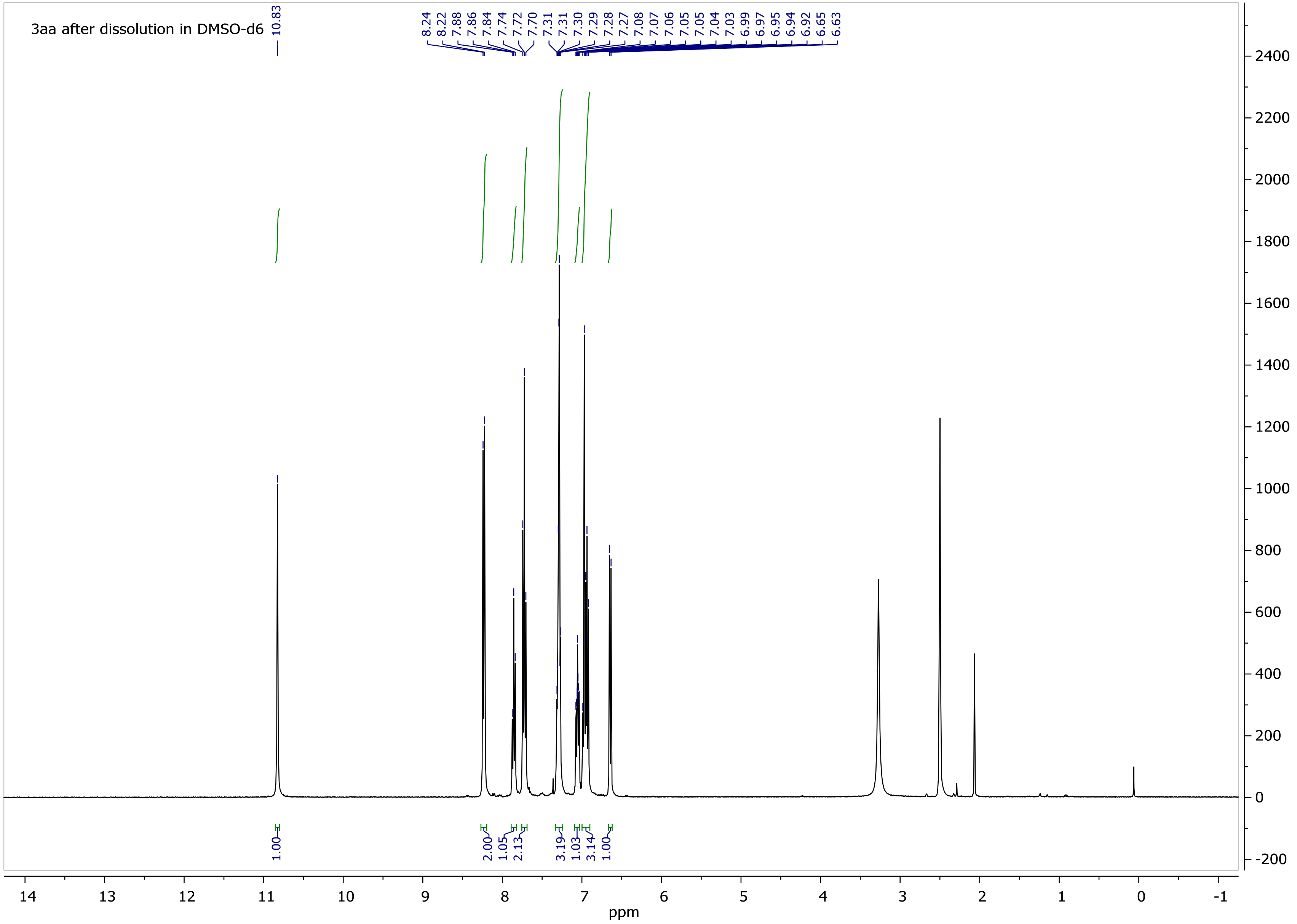

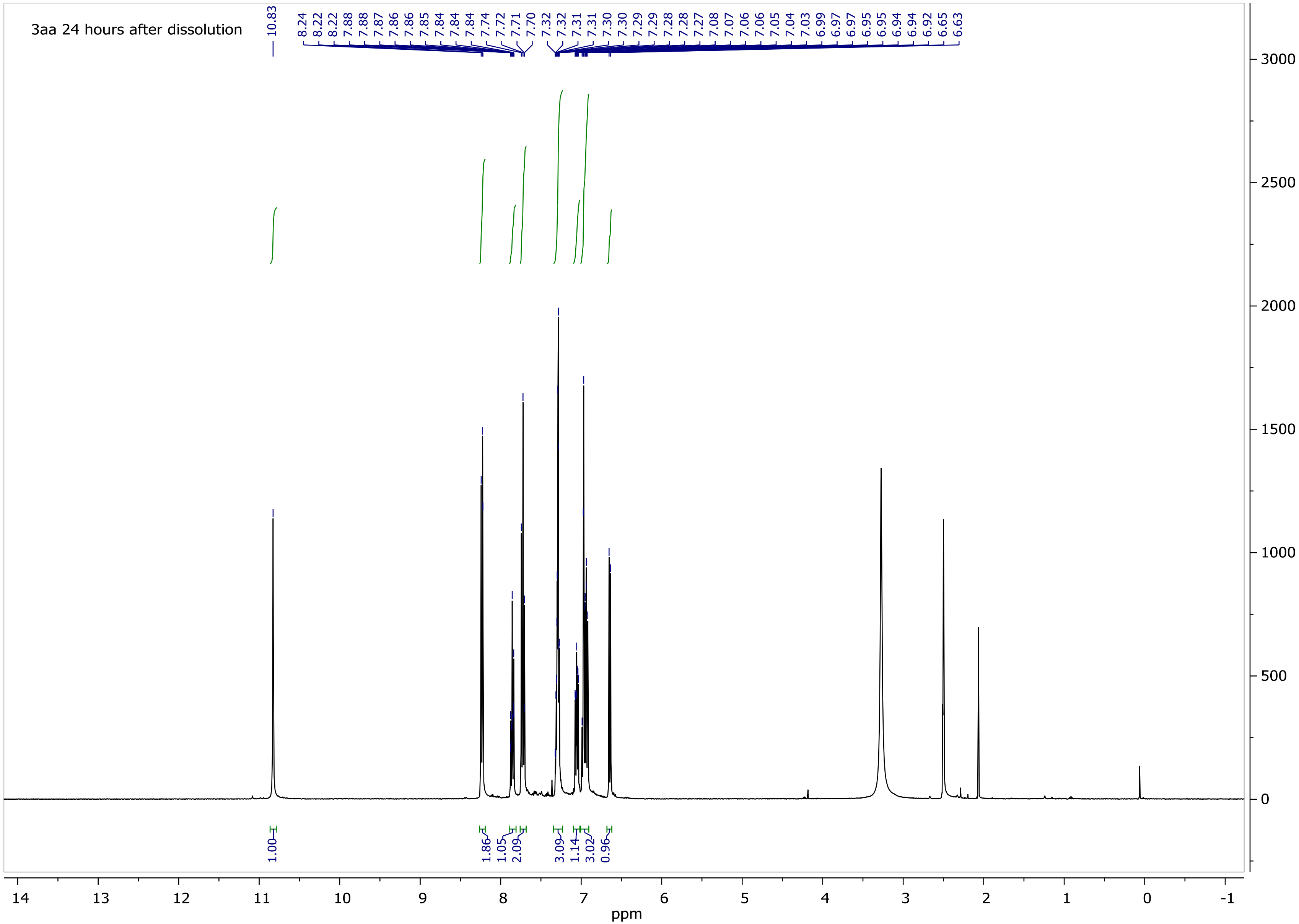
3aa 24 hours after dissolution
10.83
8.24
8.22
8.22
7.88
7.87
7.86
7.86
7.85
7.84
7.84
7.84
7.74
7.72
7.71
7.70
7.32
7.32
7.31
7.31
7.30
7.30
7.29
7.29
7.28
7.28
7.27
7.08
7.07
7.06
7.06
7.05
7.04
7.03
6.99
6.97
6.97
6.95
6.95
6.94
6.94
6.92
6.65
6.63
1.00
1.86
1.05
2.09
3.09
1.14
3.02
0.96
ppm

X-Ray crystal data of CCDC 2201614 (3aa)

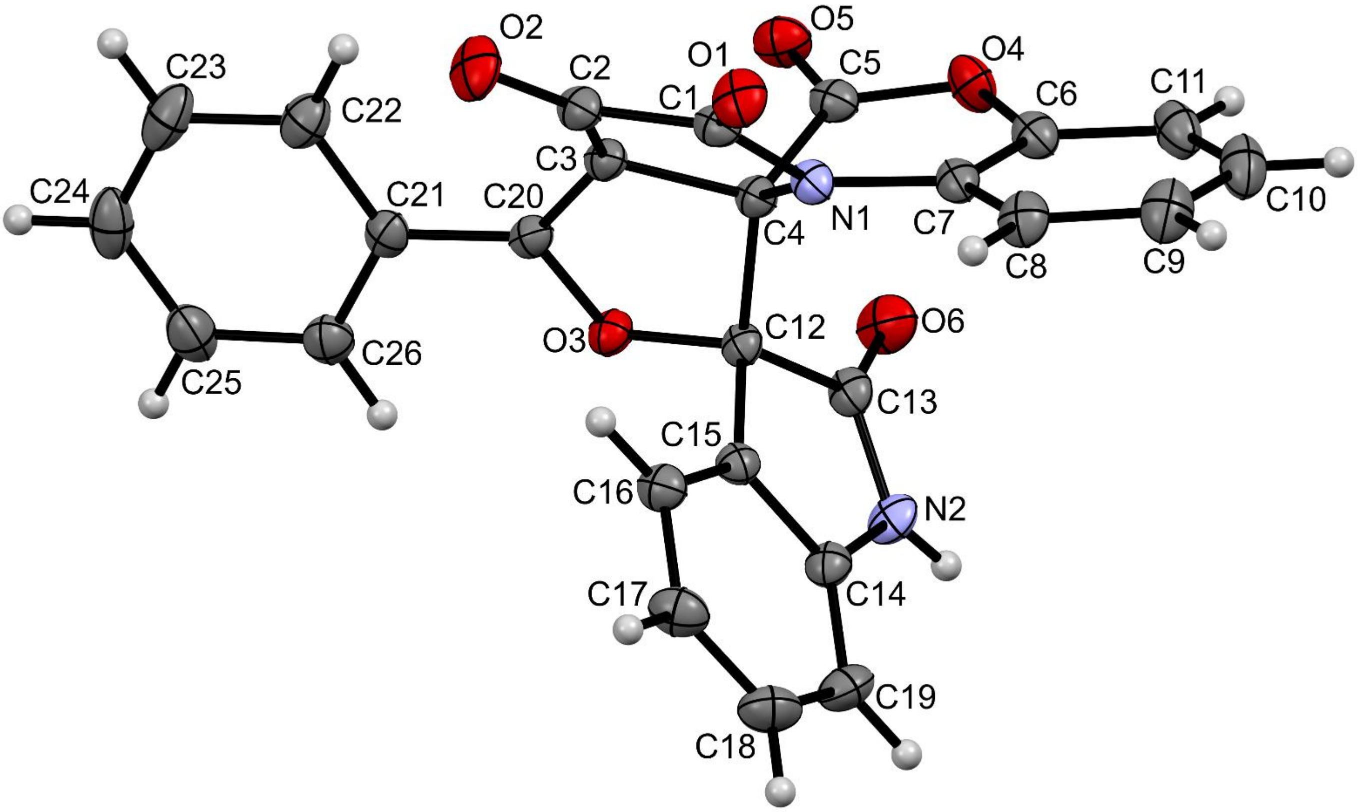

Figure S1. Molecular structure of compound **3aa** showing 30% probability amplitude displacement ellipsoids (CCDC 2201614).

Table S1. Crystal data and structure refinement for compound **3aa**.

Empirical formula	$C_{26}H_{14}N_2O_6$
Formula weight	450.39
Temperature, K	295.15
Crystal system	triclinic
Space group	P-1
a, Å	9.1925(12)
b, Å	9.5653(12)
c, Å	12.2709(16)
α, °	79.986(11)
β, °	82.628(10)
γ, °	67.316(12)
Volume, Å^3	978.1(2)
Z	2
Density (calculated), g/cm^3	1.529
Absorption coefficient, mm^{-1}	0.111
F(000)	464.0

Crystal size, mm^3	0.56 × 0.44 × 0.19
Radiation	MoKα (λ = 0.71073)
2Θ range for data collection, °	6.066 to 58.674
Index ranges	$-12 \leq h \leq 12$, $-8 \leq k \leq 13$, $-16 \leq l \leq 16$
Reflections collected	7645
Independent reflections	4485 [R_{int} = 0.0275, R_{sigma} = 0.0396]
Data/restraints/parameters	4485/0/311
Goodness-of-fit on F^2	1.040
Final R indexes [I>=2σ (I)]	R_1 = 0.0457, wR_2 = 0.1113
Final R indexes [all data]	R_1 = 0.0573, wR_2 = 0.1219
Largest diff. peak/hole, $eÅ^{-3}$	0.26/-0.23

X-Ray crystal data of CCDC 2201616 (3ab)

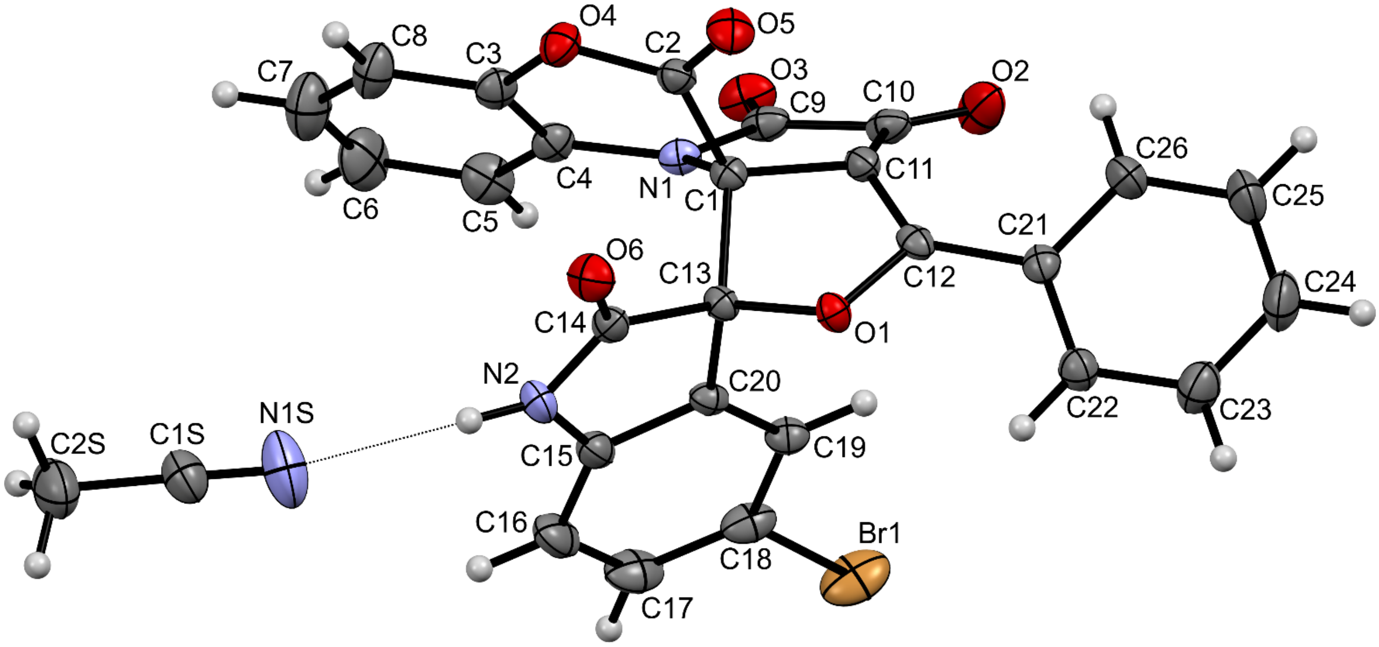

Figure S2. Molecular structure of compound **3ab** showing 30% probability amplitude displacement ellipsoids (CCDC 2201616).

Table S2. Crystal data and structure refinement for compound **3ab**.

Empirical formula	$C_{28}H_{16}BrN_3O_6$
Formula weight	570.35
Temperature, K	295.15
Crystal system	triclinic
Space group	P-1
a, Å	8.9672(17)
b, Å	11.983(3)
c, Å	13.017(2)
α, °	107.746(17)
β, °	93.660(15)
γ, °	109.614(18)
Volume, Å^3	1233.2(4)
Z	2
Density (calculated), g/cm^3	1.536
Absorption coefficient, mm^{-1}	1.717
F(000)	576.0
Crystal size, mm^3	0.55 × 0.5 × 0.4
Radiation	Mo Kα (λ = 0.71073)
2Θ range for data collection, °	5.754 to 58.83
Index ranges	$-11 \le h \le 9$, $-15 \le k \le 16$, $-17 \le l \le 16$

Reflections collected	10367
Independent reflections	5707 [R_{int} = 0.0369, R_{sigma} = 0.0656]
Data/restraints/parameters	5707/1/348
Goodness-of-fit on F^2	1.021
Final R indexes [I>=2σ (I)]	R_1 = 0.0576, wR_2 = 0.1315
Final R indexes [all data]	R_1 = 0.0935, wR_2 = 0.1578
Largest diff. peak/hole, eÅ^{-3}	0.91/-1.23

X-Ray crystal data of CCDC 2201615 (3ha)

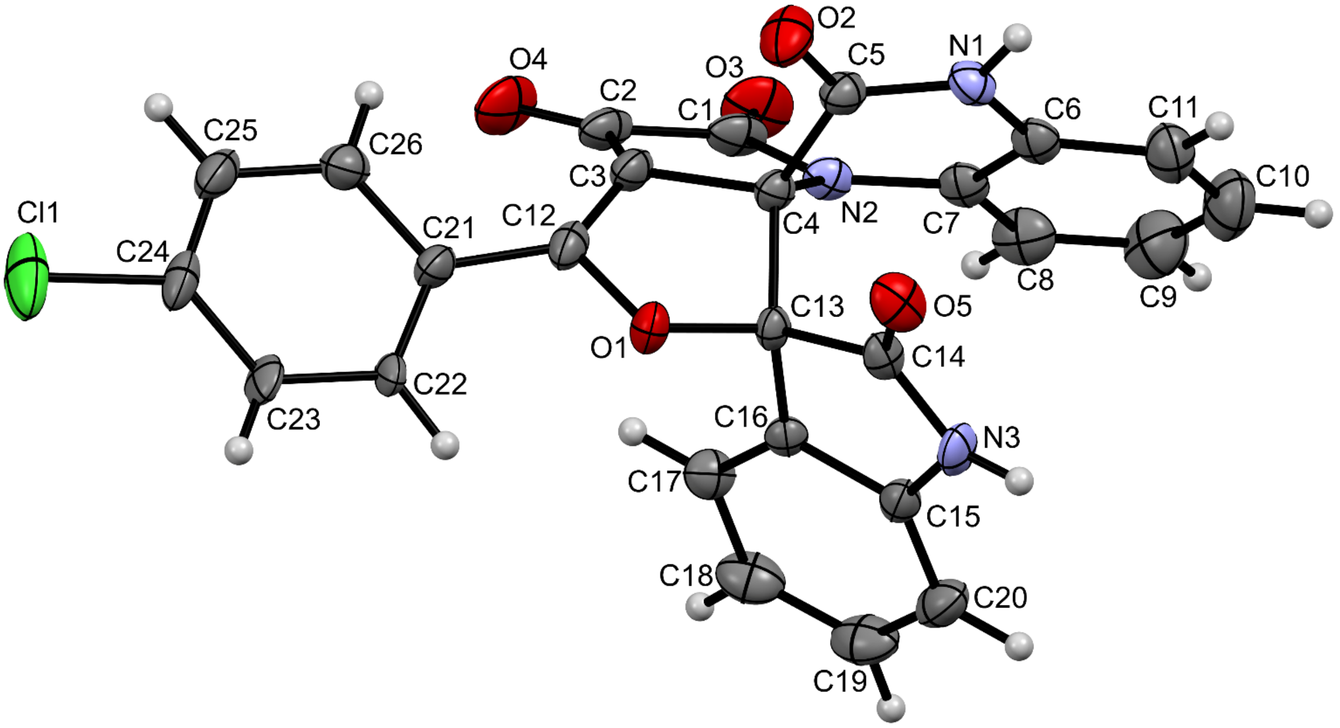

Figure S3. Molecular structure of compound **3ha** showing 30% probability amplitude displacement ellipsoids (CCDC 2201615).

Table S3. Crystal data and structure refinement for compound **3ha**.

Empirical formula	$C_{26}H_{14}ClN_3O_5$
Formula weight	483.85
Temperature, K	295.15
Crystal system	monoclinic
Space group	C2/c
a, Å	22.204(3)
b, Å	11.7801(15)
c, Å	17.325(2)
α, °	90
β, °	90.997(12)
γ, °	90
Volume, $Å^3$	4531.0(11)
Z	8
Density (calculated), g/cm^3	1.419
Absorption coefficient, mm^{-1}	0.213
F(000)	1984.0
Crystal size, mm^3	0.5 × 0.3 × 0.2
Radiation	Mo Kα (λ = 0.71073)
2Θ range for data collection, °	6.016 to 58.588

Index ranges	$-20 \leq h \leq 28$, $-10 \leq k \leq 16$, $-21 \leq l \leq 22$
Reflections collected	11513
Independent reflections	5313 [R_{int} = 0.0414, R_{sigma} = 0.0537]
Data/restraints/parameters	5313/84/384
Goodness-of-fit on F^2	1.032
Final R indexes [I>=2σ (I)]	R_1 = 0.0509, wR_2 = 0.1263
Final R indexes [all data]	R_1 = 0.0812, wR_2 = 0.1488
Largest diff. peak/hole, eÅ^{-3}	0.21/-0.26

References

1. CrysAlisPro, Agilent Technologies, Version 1.171.37.33 (release 27-03-2014 CrysAlis171 .NET).
2. Dolomanov O. V., Bourhis L. J., Gildea R. J, Howard J. A. K., Puschmann H. *J. Appl. Cryst.*, **2009**, *42*, 339.
3. Palatinus L., Chapuis G. *J. Appl. Cryst.*, **2007**, *40*, 786.
4. Bourhis L. J., Dolomanov O. V., Gildea R. J., Howard J. A. K., Puschmann H. *Acta Crystallogr., Sect. A: Found. Adv.*, **2015**, *71*, 59.
5. Sheldrick G. M. *Acta Crystallogr., Sect. C: Struct. Chem.*, **2015**, *71*, 3.
6. (a) Bozdyreva K. S., Smirnova I. V., Maslivets A. N. *Russ. J. Org. Chem.*, **2005**, *41*, 1081; (b) Mashevskaya I. V., Mokrushin I. G., Bozdyreva K. S., Maslivets A. N. *Russ. J. Org. Chem.*, **2011**, *47*, 253; (c) Maslivets A. N., Mashevskaya I. V., Smirnova L. I., Krasnykh O. P., Shurov S. N., Andreichikov Yu. S. *Zh. Org. Khim.*, **1992**, *28*, 2545; (d) Stepanova E. E., Babenysheva A. V., Maslivets A. N. *Russ. J. Org. Chem.*, **2011**, *47*, 937; (e) Mashevskaya I. V., Mokrushin I. G., Bozdyreva K. S., Maslivets A. N. *Russ. J. Org. Chem.*, **2011**, *47*, 253, (a) Tret'yakov N. A., Dmitriev M. V., Maslivets A. N. *Russ. J. Org. Chem.*, **2020**, *56*(8), 1367.
7. Marek L., Kolman L., Váňa J., Svoboda J., Hanusek J. *Beilstein J. Org. Chem.*, **2021**, *17*(1), 527.
